# Oxygen tank for synergistic hypoxia relief to enhance mitochondria-targeted photodynamic therapy

**DOI:** 10.1186/s40824-022-00296-0

**Published:** 2022-09-22

**Authors:** Xianghui Li, Haoran Wang, Zhiyan Li, Dandan Li, Xiaofeng Lu, Shichao Ai, Yuxiang Dong, Song Liu, Jinhui Wu, Wenxian Guan

**Affiliations:** 1 Department of Gastrointestinal Surgery, Affiliated Nanjing Drum Tower Hospital, Nanjing University Medical School, Nanjing, 210008 China; 2 State Key Laboratory of Pharmaceutical Biotechnology, Medical School and School of Life Science, Nanjing University, Nanjing, 210093 China; 3 Department of Pharmacology, School of Pharmacy, Nanjing University of Chinese Medicine, Nanjing, 210023 China; 4 First Clinical Medical College of Nanjing Medical University, Nanjing, 210029 China; 5 Chemistry and Biomedicine Innovation Center, Nanjing University, Nanjing, 210023 China; 6 Jiangsu Key Laboratory for Nano Technology, Nanjing University, Nanjing, 210093 China

**Keywords:** Organelle targeted therapy, Mitochondrial respiratory inhibition, PDT, Artificial red blood cells, Synergistic oxygen modulation, Tumor hypoxia

## Abstract

**Background:**

Mitochondria play an essential role in cellular redox homeostasis maintenance and meanwhile serve as an important target for organelle targeted therapy. Photodynamic therapy (PDT) is a promising strategy for organelle targeted therapy with noninvasive nature and highly spatiotemporal selectivity. However, the efficacy of PDT is not fully achieved due to tumor hypoxia. Moreover, aerobic respiration constantly consumes oxygen and leads to a lower oxygen concentration in mitochondria, which continuously limited the therapeutic effects of PDT. The lack of organelle specific oxygen delivery method remains a main challenge.

**Methods:**

Herein, an Oxygen Tank is developed to achieve the organelle targeted synergistic hypoxia reversal strategy, which not only act as an oxygen storage tank to open sources and reduce expenditure, but also coated with red blood cell membrane like the tank with stealth coating. Within the oxygen tank, a mitochondrion targeted photosensitizer (IR780) and a mitochondria respiration inhibitor (atovaquone, ATO) are co-loaded in the RBC membrane (RBCm) coated perfluorocarbon (PFC) liposome core.

**Results:**

Inside these bio-mimic nanoparticles, ATO effectively inhibits mitochondrial respiration and economized endogenous oxygen consumption, while PFC supplied high-capacity exogenous oxygen. These Oxygen modulators reverse the hypoxia status in vitro and in vivo, and exhibited a superior anti-tumor activity by mitochondria targeted PDT via IR780. Ultimately, the anti-tumor effects towards gastric cancer and colon cancer are elicited in vivo.

**Conclusions:**

This oxygen tank both increases exogeneous oxygen supply and decreases endogenous oxygen consumption, may offer a novel solution for organelle targeted therapies.

**Supplementary Information:**

The online version contains supplementary material available at 10.1186/s40824-022-00296-0.

## Introduction

Sub-cellular, or organelle-targeted therapy, not only increases the spatiotemporal selectivity but also improves therapeutic efficacy [1]. Organelles, including mitochondria, lysosomes, and endoplasmic, play a vital role in cell morphology and function maintaining, but lead to cell dysfunction, apoptosis, and death once damaged. Among these important organelles, mitochondria are often thought of as an important therapeutic target [2, 3]. Mitochondria produce adenosine triphosphate via aerobic respiration. But they are sensitive to reactive oxygen species (ROS) and involved in redox homeostasis [4], apoptosis [5, 6], and necrosis pathways regulating [7]. During the last few decades, tremendous strategies for mitochondria-mediated damage therapies have been proposed, such as energy metabolism interference [8], ROS generation [9–11], and mitophagy inducing [12, 13]. These designed mitochondria-targeted therapies exhibited superior strengths and decreased systemic toxicity. Of which, photodynamic therapy (PDT) is a novel and promising strategy with noninvasive nature and spatiotemporal selectivity [14], for that the ROS with a short half-life can only act on the production site (< 20 nm), much smaller than a cell size [15]. However, the efficacy of organelle-targeted PDT has been severely limited due to the hypoxia status in most solid tumors, thus oxygen delivery is necessary for PDT enhancement [16, 17].

Enormous efforts were devoted to alleviating the tumor hypoxia, including physical strategies, chemical strategies, and biological strategies. Physical strategies are achieved by oxygen delivery via high-oxygen-solubility agents such as PFC [18, 19]. Limited by the amount of dissolved oxygen in PFC, hypoxia status in the tumor can only be partially alleviated. Chemical strategies mean catalytic oxygen production from chemical molecules [20, 21]. Though the concentration of reactive substances (such as H_2_O_2_) in the tumor site is higher than in normal tissue, it is still not enough (< 50 μM) for oxygen generation and is not satisfactory for PDT consumption. Biological strategies include blood flow improvement and in situ oxygen production by microorganisms [22–24]. Nevertheless, because of the abnormal blood vessels and the limited survival time of microorganisms, the oxygen supplied is insufficient. Meanwhile, owing to a short coverage range, ROS generated by PDT only acts on targeted organelles but does not damage distant structures or proteins. The lack of organelle-specific oxygen delivery method remains the main challenge [25–27]. At the same time, PDT consumed oxygen to generate ROS, which caused severe hypoxia within mitochondria, where a lower oxygen concentration had exhibited due to aerobic respiration, and ultimately decrease PDT efficacy to some degree.

Mitochondria constantly consume oxygen via the phosphorylation (OXPHOS) metabolic pathway and produce energy for cell survival. OXPHOS blocking, one of the most efficient means to intervene in mitochondria-mediated metabolism, involves inhibiting mitochondrial respiratory chain complexes to induce multilevel mitochondrial disorders. It is reported that OXPHOS inhibition leads to decreased mitochondria membrane potential, unstable mitochondrial morphology, disturbing mitochondrial respiration distribution, lessen ATP production, and strengthened ROS generation [1]. Especially, complex III on the electro-transport chain (ETC) is regarded as the major site of ROS generation in mitochondria. Once interfered with by the inhibitor, complex III releases ROS to cytoplasmic [28, 29], leading to organelles being damaged and cell dysfunction. Thus, OXPHOS has been regarded as an important target for cancer treatment. More importantly, by inhibiting electron transport in OXPHOS, the oxygen consumption by mitochondria decreased due to aerobic respiration blocking. As a result, the oxygen level around mitochondria increased and ultimately resulted in PDT effect enhancement [30].

**Scheme 1:**
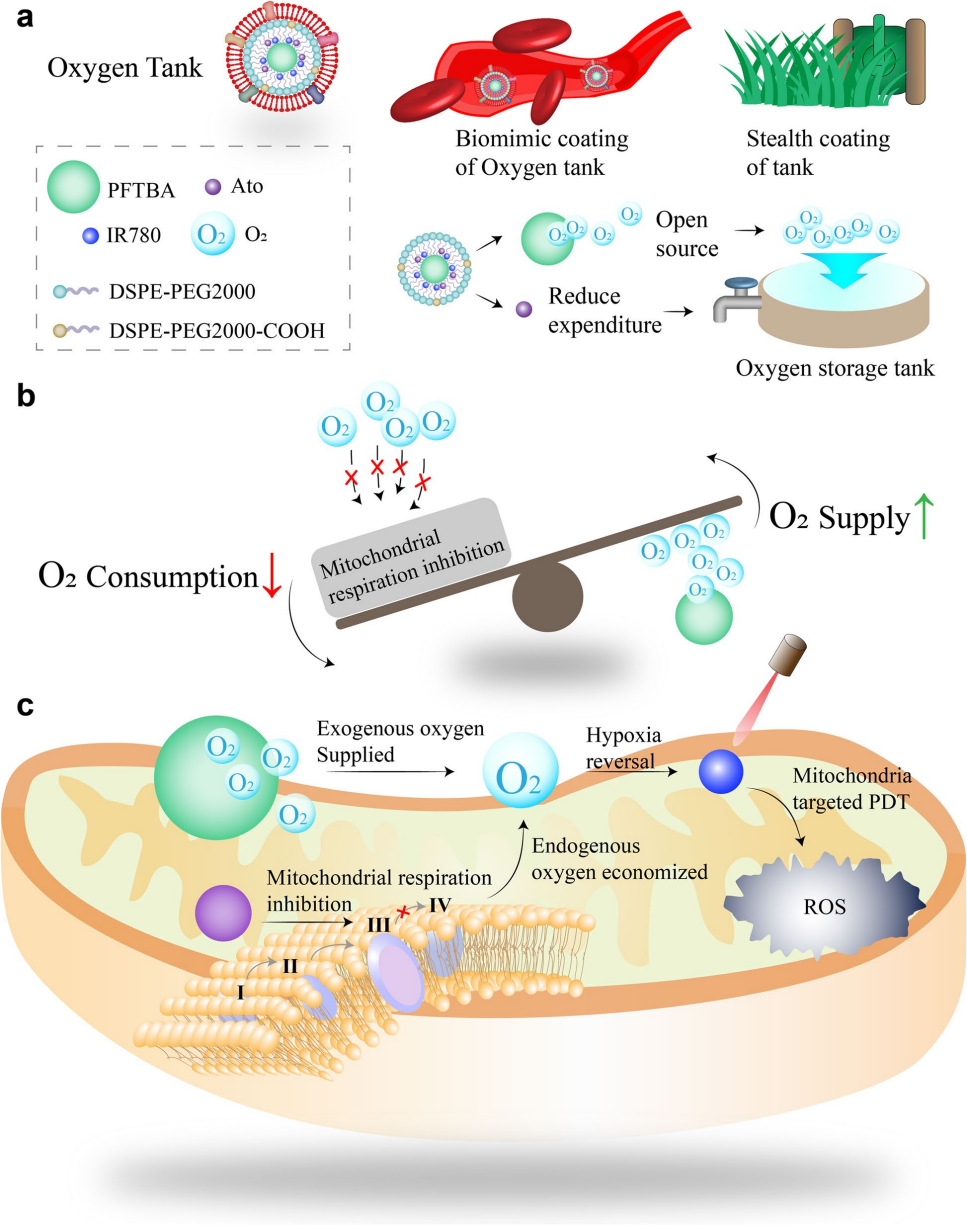
Schematic illustration of the design, synergistic hypoxia reversal function, and therapeutic functions of the Oxygen Tank. a) Design illustration of the Oxygen Tank. On the one hand, the biomimetic coating of the Oxygen Tank is similar to the stealth coating of a battle tank; On the other hand, the Oxygen Tank opened source and reduced the expenditure of oxygen as a gas tank. b) Oxygen Tank reduced oxygen consumption by mitochondria respiration inhibition and increased oxygen supply by PFC to achieve synergistic hypoxia regulation. c) Such synergistic hypoxia reversal and Mt-PDT strategy simultaneously supplied exogenous oxygen and inhibited endogenous oxygen consumption to manipulate the tumor hypoxia microenvironment, and ultimately attack the mitochondria of tumor cells

Herein, we developed a nano-enabled approach for enhanced mitochondria-targeted PDT (Mt-PDT) (Scheme 1). This multi-level antitumor therapy was achieved through exogenous O_2_ delivery, endogenous hypoxia inhibition, mitochondria dysfunction, and Mt-PDT by artificial red blood cells (RBCs), named Oxygen tank. Specifically, atovaquone (ATO) and a kind of photosensitizers, 2-[2-[2-chloro-3-[(1,3-dihydro3,3-dimethyl-1-propyl-2 h-indol-2-ylidene) ethylidene]-1-cyclohexen-1yl] ethenyl]-3,3-dimethyl-1-propylindolium iodide (IR780), were co-loaded in a core-shell structure liposome with a perfluorocarbon (PFC) core and red blood cell membrane (RBCm) coat. ATO, a mitochondrial ETC complex III inhibitor, is believed to reduce O_2_ consumption and increase ROS generation [28, 29] and has been approved by the United States Food and Drug Administration (FDA) to treat malaria. IR780 is a cationic lipid-soluble dye featured with mitochondria-targeted ability and can be used as a PDT agent [31, 32]. In vitro and in vivo evaluations were conducted in this work to verify this synergistic hypoxia relief strategy for mitochondria inhibition and damage amplified PDT. We believe that the bio-mimic nanoparticles serve as oxygen storage tanks via exogenous O_2_ delivery, endogenous hypoxia inhibition, and provide an effective method for mitochondria dysfunction and Mt-PDT. It may also be a novel hypoxia modulator for solid tumors such as gastric cancer and colon cancer, which is essential for combatting treatment resistance, not limited to PDT.

## Results

### Characterization of oxygen tank

**Fig. 1:**
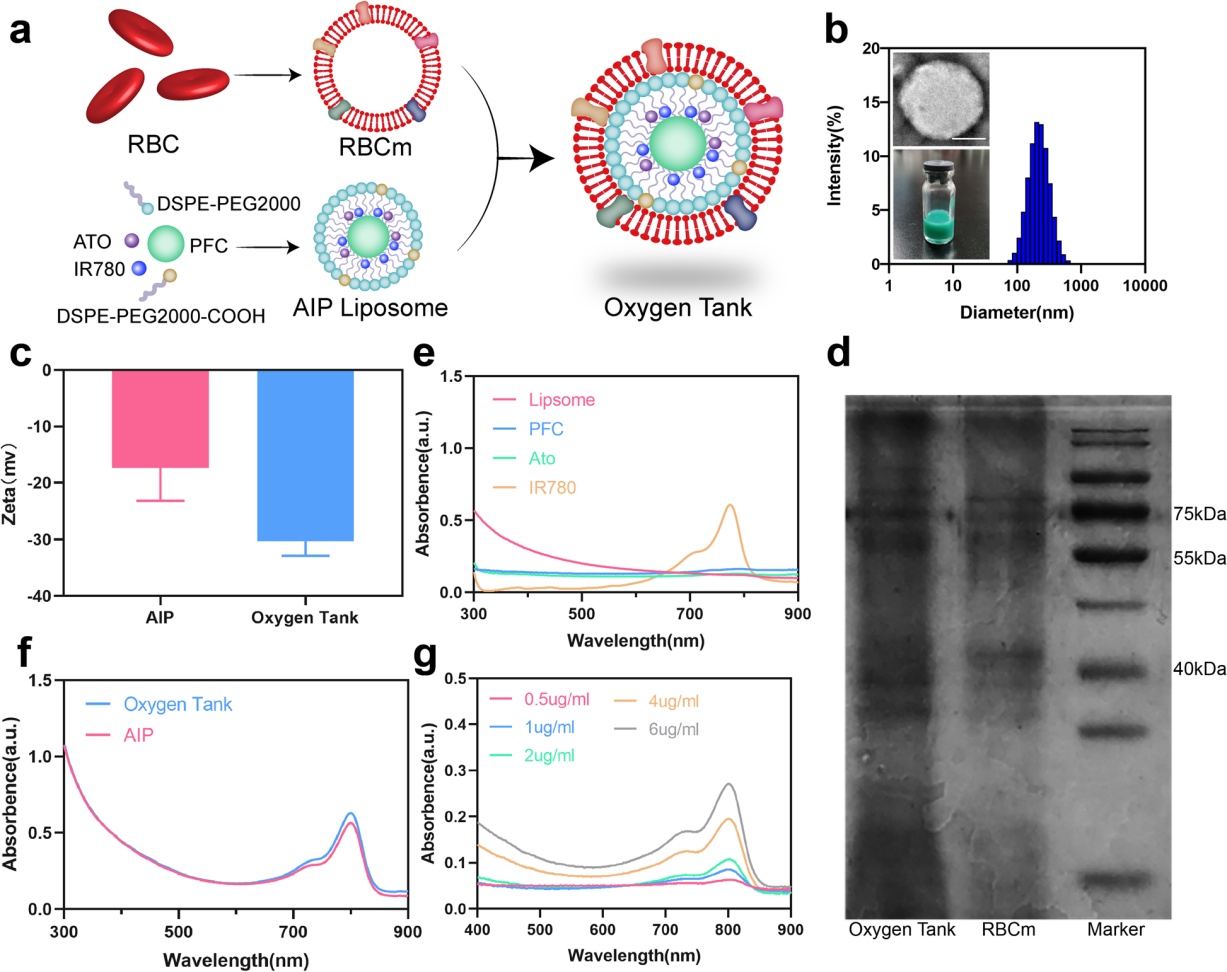
Characterization of the Oxygen Tank. **a** Schematic illustration of the design and synthesis of the Oxygen Tank. **b** Hydrodynamic diameters of AIP. Inset shows photographs and TEM images of these NPs. The scale bars are 100 nm. **c** Zeta potential of AIP NPs and Oxygen Tank. **d** Result of SDS-PAGE analysis to test whether similar protein brands exhibited in Oxygen Tank compared to RBCm. **e** UV-vis spectra of the free liposome, PFC, ATO, and IR780. **f** UV vis spectra of the Oxygen Tank and AIP NPs in PBS. **g** UV-vis spectra of Oxygen Tank in different concentrations (0.5 μg/ml, 1 μg/ml, 2 μg/ml, 4 μg/ml, and 6 μg/ml)

RBCm@Ato-IR780-PFC liposomes (oxygen tanks) were synthesized according to Scheme 1. Briefly, a photosensitizer (IR780) and a commonly used malaria drug (ATO) were packaged in liposomes with a PFC core and an RBCm coat (Fig. 1a). As shown in Fig. 1b and Additional file 1: Fig. S1a, the Oxygen tank and ATO-IR780-PFC liposomes (AIP) both performed a homogeneously dispersed spherical morphology. We used DLS to measure the hydrodynamic sizes of these NPs. The mean hydrodynamic sizes of Oxygen tank and AIP were 238.00 and 214.73 nm respectively. Such transformation of size may indicate that RBCm, whose thickness is about 5 to 10 nm, has been successfully coated on AIP NPs [33]. Transmission electron microscopy (TEM) images and photographs of these NPs were shown inset and further revealed that RBCm had been completely coated on AIP NPs. Zeta potentials of AIP and Oxygen tank were − 17.34 mV and − 30.29 mV, respectively, which attributed to the negative surface charge of pure RBCm (Fig. 1c) [34]. As indicated in vitro safety analysis, no statistical significance was observed in Oxygen tank at the concentration lower than 8 μg/mL (Additional file 1: Fig. S1b). Additionally, sodium salt–polyacrylamide gel electrophoresis (SDS-PAGE) was conducted to examine the protein of compositions of RBCm and Oxygen tank (Fig. 1d). The same protein brands verify the existence of RBCm proteins in the Oxygen tank sample. Figure 1e demonstrated the absorption spectra IR780, ATO, PFC and liposome. The typical absorption peak of IR780 appeared at 774 nm. Figure 1f indicated that ATO, IR780, PFC, and liposome were successfully loaded into AIP and Oxygen tank. Moreover, high-performance liquid chromatography was performed and the drug loading and entrapment efficiency were 76.7 and 0.94%, respectively (Additional file 1: Fig. S2, Fig. S3, Table S1, Table S2). The drug loading and entrapment efficiency of PFC were 82.0 and 19.6%, respectively (Additional file 1: Fig. S4). Different UV-vis absorption spectra of Oxygen tank at different concentrations were detected and the concentration curve was provided (Fig. 1g and S1c). The diameters of AIP and Oxygen tank in PBS were detected every 12 h by DLS, and they both maintained stable within 96 h (Additional file 1: Fig. S1d).

### Synergistic hypoxia reversal in vitro

**Fig. 2:**
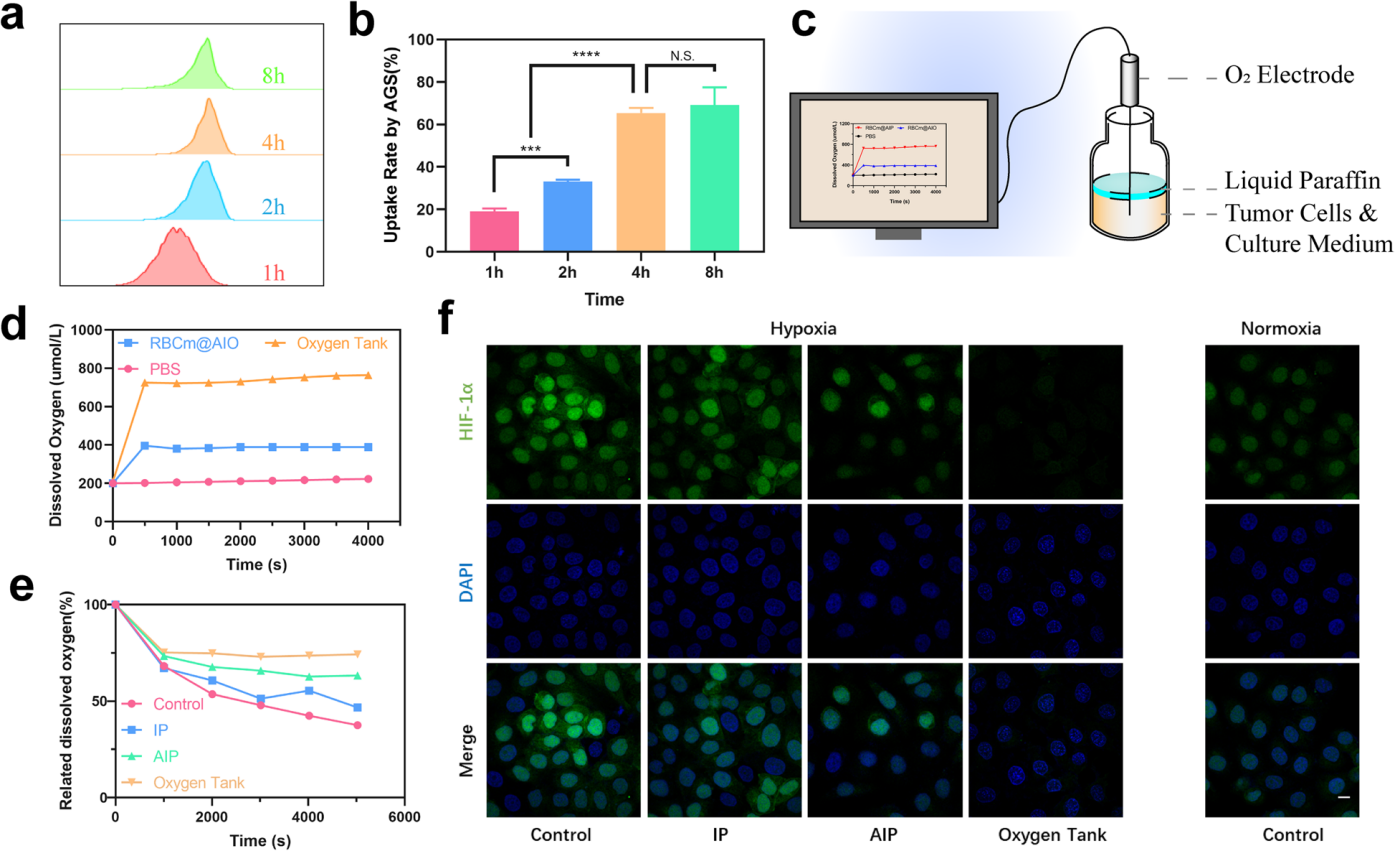
Synergistic hypoxia reversal by oxygen supplied and consumption reduced. **a** The uptake behavior of the Oxygen Tank by AGS cells in different time points (1 h, 2 h, 4 h, and 8 h) is determined by flow cytometry. **b** Quantification result of the flow cytometry evaluation. **c** Schematic illustration of instrument for dissolved oxygen measurement. **d** Oxygen release curves of different NPs (PBS, RBCm@AIP NPs, and Oxygen Tank). **e** The dissolved oxygen curves of the culture medium within AGS cells after different treatments (PBS, IP NPs, AIP NPs, and Oxygen Tank). **f** Confocal fluorescence images of Hif-1α staining of AGS cells after different treatments (PBS in hypoxia condition, IP NPs in hypoxia condition, AIP NPs in hypoxia condition, Oxygen Tank in hypoxia condition, and PBS in normoxia condition). The scale bar is 20 μm

As revealed in Fig. 2a and b, the result of flow cytometry demonstrated that the uptake behavior of Oxygen tank NPs by AGS cells was time-dependent. Thus 4 h may be the optimized time for co-incubation treatment. To assess the oxygen supplied and economized by the Oxygen tank, the oxygen electrode-based instrument was established to monitor the real-time O_2_ level in liquid paraffin sealed system (Fig. 2c). To verify the oxygen supply by PFC, oil nanoparticles (RBCm@ATO-IR780-Olive oil, RBCm@AIO) were prepared by replacing PFTBA with olive oil as controls [35]. As shown in Fig. 2d, we found a remarkable dissolved oxygen increase in Oxygen tank solution (~ 500 μM) compare to PBS. Though RBCm@AIO solution also increased dissolved oxygen (~ 250 μM), the high-capacity oxygen ability of PFC was revealed.

Additionally, we expected Oxygen tanks are capable of reducing oxygen consumption via OXPHOS inhibition by ATO. AGS cells were incubated with different culture mediums (control, IR780-PFTBA (IP) NPs, AIP NPs, and Oxygen tank). As revealed in Fig. 2e, without the introduction of PFC and ATO, the dissolved oxygen in a culture medium of AGS cells gradually decreased with the extending of incubation time, demonstrating a continued oxygen consumption. With the participation of IP NPs, hypoxia was slightly relieved. However, because of the high consumption of cancer cells, dissolved oxygen gradually decreased. Nevertheless, with the introduction of ATO in the AIP group and the Oxygen tank group, oxygen consumption was slowed significantly. Especially, in the Oxygen tank group, dissolved oxygen stayed stable for more than 1 h. Undoubtedly, Oxygen tank increased oxygen supplied and reduced cellular oxygen consumption, thus relieving tumor hypoxia.

Furthermore, hypoxia-inducible factor-1α (HIF-1α) staining was conducted to verify that the Oxygen tank reversed tumor cell hypoxia (Fig. 2f and Additional file 1: Fig. S5). Hypoxia existed in various solid tumors due to rapid proliferation and hypermetabolism. Strong green fluorescence was detected in the control group to simulate a hypoxia microenvironment. Comparatively, with the introduction of PFC, moderate fluorescence was observed in the IP group. Moreover, AGS cells in the AIP group exhibited a weak fluorescence while the Oxygen tank group showed the weakest fluorescence (*p* < 0.0001). Based on the results above, we verify the assumption that Oxygen tank NPs could both increase exogenous oxygen supply and inhibit endogenous oxygen consumption to overcome hypoxia in tumor cells. This synergistic exogenous and endogenous oxygen modulated strategy through the introduction of PFC and ATO was first reported. We hope this synergistic modulator could solve the problem of tumor hypoxia in PDT and thus combat the unfavorable prognosis of tumors.

### Long circulation and enhanced accumulated in the tumor by oxygen tank

**Fig. 3:**
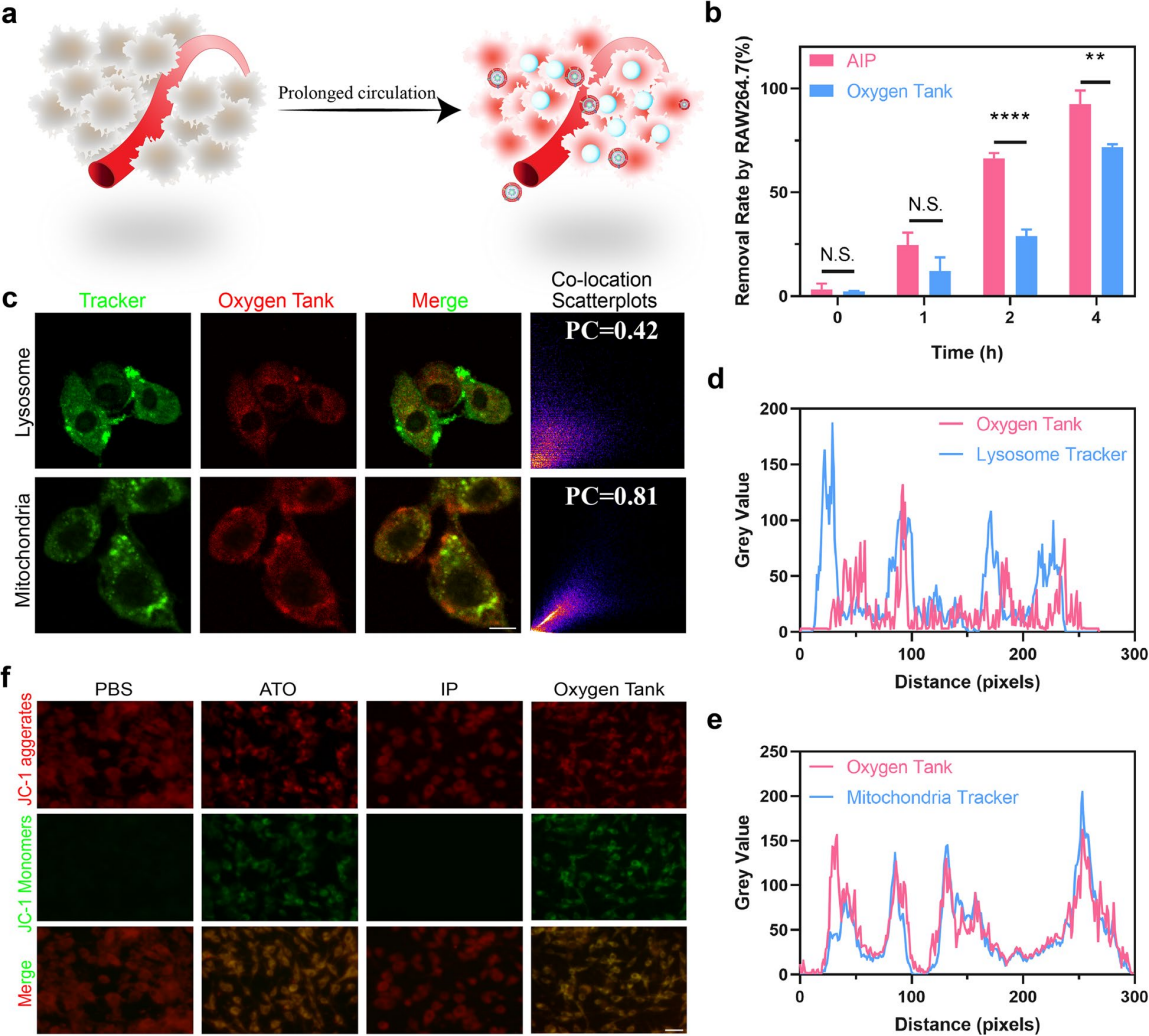
Prolonged circulation and mitochondria-targeted ability of Oxygen Tank. **a** Schematic illustration of the prolonged circulation by RBCm-coating NPs. **b** The clearance rate of the Oxygen Tank is determined by flow cytometry. RAW264.7 cells were treated with the Oxygen Tank or AIP for different lengths of time (0 h, 1 h, 2 h, and 4 h). **c** Subcellular localization of Oxygen Tank compared to lysosome and mitochondria trackers determined by CLSM. The scale bar is 10 μm. PC means the Pearson Correlation coefficient. **d**. Colocalization analysis of Oxygen Tank in AGS cells with lysosome tracker. **e** Colocalization analysis of Oxygen Tank in AGS cells with mitochondria tracker. **f** Mitochondria potential evaluation by JC-1 after AGS cells treated with PBS, ATO, IP NPs, and Oxygen Tank NPs. Red signals (JC-1 aggregates) suggested a normal polarized mitochondrial membrane. Green signals (JC-1 monomers) suggested an abnormal depolarized mitochondrial membrane. The scale bar is 50 μm

Accumulating evidence suggested that the membrane components on the surface of RBCs help them survive in macrophages [36]. We expected that the Oxygen tank inherits the anti-phagocytosis capability from RBCm coating (Fig. 3a). Oxygen tank and uncoated AIP (IR780, 8 μg/ml) were incubated with mouse macrophage cells (RAW264.7) for 1, 2, and 4 h, respectively. After co-incubation, cells were washed with PBS 3 times to remove attached nanoparticles on the cell surface and then evaluated by flow cytometry (Fig. 3b). Results showed that AIP coated with RBCm reduces the removal rate by RAW264.7 significantly, indicating that RBCm coating can effectively reduce immune clearance due to the components in the RBCm.

Next, we compared the cellular uptake of the Oxygen tank and AIP in tumor cells. As revealed in Additional file 1: Fig. S6a, the results of flow cytometry demonstrated that both Oxygen tank and AIP were similar in cellular uptake by AGS cells in 1 h, 2 h, and 4 h. To further confirm this result, another cancer cell line CT26 was evaluated by flow cytometry (Additional file 1: Fig. S6b). Similar results indicated the uptake behavior was not affected by RBCm coating.

Longer blood circulation time of nanoparticles contributed to tumor accumulation, we next examined the tumor distribution of Oxygen tank and AIP on tumor-bearing mice (Additional file 1: Fig. S6c). The fluorescence signals of IR780 in vivo were performed at different time points after intravenous injection. It was found that the Oxygen tank achieved higher fluorescence signals of IR780 in the tumor site, which reached the maximum at 24 h after injection. Though fluorescence signals of AIP increased gradually, the signals are much lower than the Oxygen tank. We could conclude that RBCm coating on Oxygen tank helps them accumulate in the tumor site.

### The mitochondria-targeted capability of oxygen tank and mitochondria disorder evaluation

According to our previous report, IR780, a lipophilic cationic dye, could specifically bind with mitochondria [37]. To identify our assumption that the Oxygen tank NPs could combine to mitochondria, we compared the subcellular localization of designated organelles and NPs in vitro. As revealed in Fig. 3c, the different subcellular localization was observed between the red signals from the Oxygen tank as the green signals of lysosomes. However, the red signals performed almost the same subcellular localization as the green signals from mitochondria. The Pearson Correction (PC) coefficient of IR780 and lysosome was 0.42, while PC of IR780 and mitochondria was 0.81, according to the co-location scatterplots. Furthermore, colocalization analysis of the Oxygen tank with the lysosome tracker demonstrated a different trend (Fig. 3d), while the mitochondria tracker exhibited a similar trend (Fig. 3e). Therefore, we concluded that the Oxygen tank exhibited mitochondria-targeted intracellular localization features. Thus, the strategy of mitochondria-targeted PDT could be realized.

ATO inhibits the respiratory chain electron transfer, which supports the preservation of membrane potential, and ultimately spares oxygen [38]. The cyanine dye JC-1 was used to assess the changes in mitochondrial membrane potential. As revealed in Fig. 3f, normal cells with high mitochondrial membrane potential were observed as the dominant population of red signals (JC-1 aggregate). Abundant green signals (JC-1 monomers) in the ATO group suggested a depolarized mitochondrial membrane because the mitochondria respiratory chain electron transfer was blocked. As expected, cancer cells in the IP group exhibited a normal mitochondrial membrane potential, whereas a depolarized mitochondrial potential was witnessed in cancer cells that treated with Oxygen tanks.

### In vitro antitumor PDT was enhanced by mitochondria-targeted oxygen tanks

**Fig. 4:**
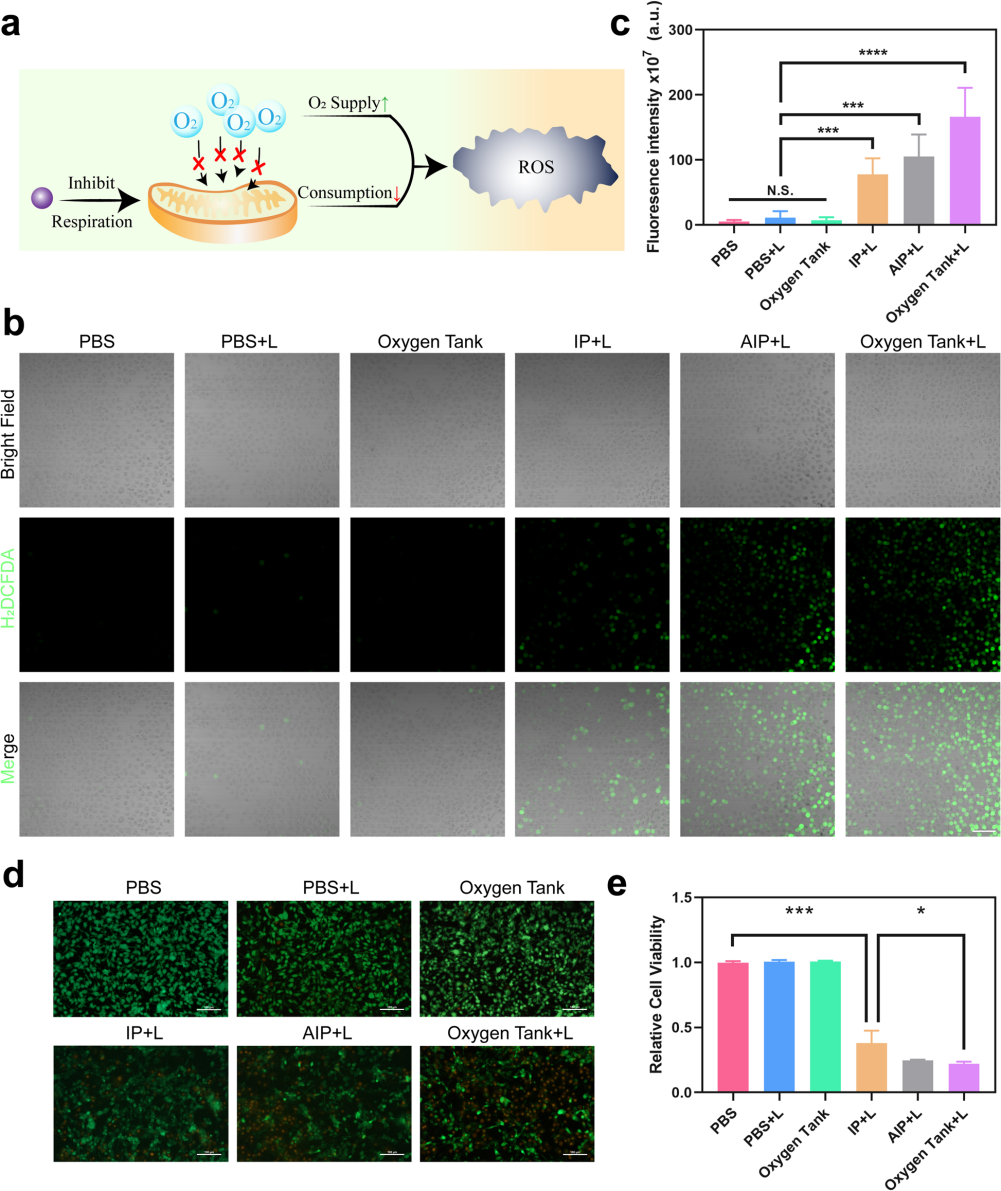
Amplified PDT by synergistic hypoxia reversal strategy. **a** Schematic illustration of the synergistic hypoxia reversal strategy, which both increased oxygen supply and decreased oxygen consumption and ultimately enhanced PDT efficacy. **b** and **c** CLSM images and fluorescence intensity quantification of ROS generation in AGS cells after different treatments (PBS, PBS with laser, Oxygen Tank, IP NPs with laser, AIP NPs with laser, and Oxygen Tank with laser). Green fluorescence stained by H2DCFHDA depicted ROS. The scale bar is 100 μm. **d** CLSM images of AGS cells after different treatments (PBS, PBS with laser, Oxygen Tank, IP NPs with laser, AIP NPs with laser, and Oxygen Tank with laser) determined by Calcein-AM/Propidium iodide double stain kit. Viable cells were stained green with Calcein-AM, and dead cells were stained red with PI. The scale bar is 100 μm. **e** Relative cell viability determined by CCK-8 kit (*n* = 4). Data are shown as mean ± SD. *p < 0.05, **p < 0.01, ***p < 0.001

After proving the hypoxia reversal and mitochondria-targeted capability of Oxygen tanks, the Mt-PDT effect of such NPs was further monitored in vitro (Fig. 4a). H_2_DCFDA was used to detect the ROS generation among different groups (Fig. 4b and c). In the PBS, PBS + laser, and Oxygen tank groups, few green signals were observed. A moderate level of ROS generation was witnessed in IP + Laser group, in which PFC offered oxygen for ROS generation. Strong green signals in AIP + laser and Oxygen tank group indicated that the intervention of mitochondrial respiration by ATO exhibited superior ROS generation via Mt-PDT.

As shown in Calcein-AM/Propidium iodide double staining results, most of the living cells (green) were observed in the IP + laser group, indicating less cytotoxicity without mitochondria inhibition by ATO (Fig. 4d, Additional file 1: Fig. S7). However, the percentage of living cells in the Oxygen tank + laser group, was significantly less than that in the IP group, indicating the PDT effect amplified by the mitochondria-targeted strategy.

CCK-8 kit was used to further detect the curative effects of the Oxygen tank with 808 nm laser irradiation in vitro. According to the results in Fig. 4e, cells in PBS, PBS + laser, and Oxygen tank groups exhibited almost no cytotoxicity. Nevertheless, the relative cell viability in IP + laser, AIP + laser, and Oxygen tank +laser was 38.0, 24.7, and 22.1%, respectively. we could conclude that Oxygen tank not only exhibited a superior biosafety property in vitro, but also demonstrated a powerful PDT effect against AGS cells, via oxygen supply by PFC, mitochondrial respiration inhibition by ATO, and mitochondria target capability of IR780.

### Biodistribution of oxygen tanks in vivo

Featured with excellent NIR imaging property from IR780 [39], the Oxygen tank could be tracked by real-time NIR fluorescence images in vivo for biodistribution analysis. The NIR images at different time points of tumor-bearing mice were shown in Additional file 1: Fig. S8a and Fig. S8c. The fluorescence signal was firstly detected at tumor site by 8 h post-injection, while the peak value was witnessed 24 h after injection.

To further evaluate the biodistribution of the Oxygen tank in major organs, ex vivo fluorescence images were shown in Additional file 1: Fig. S8b. Due to the RBCm coating, the fluorescence intensity was markedly enhanced in tumor tissue rather than the liver or renal [40]. Additionally, weak fluorescence was detected in other major organs including the spleen. The fluorescence signal intensity from tumor tissue was remarkedly higher than that of other collected organs (Additional file 1: Fig. S8d). The above results indicated that Oxygen tanks could be used in fluorescence imaging as a monitor for tumor prognosis and treatment.

### In vivo anti-hypoxia and antitumor efficacy

**Fig. 5:**
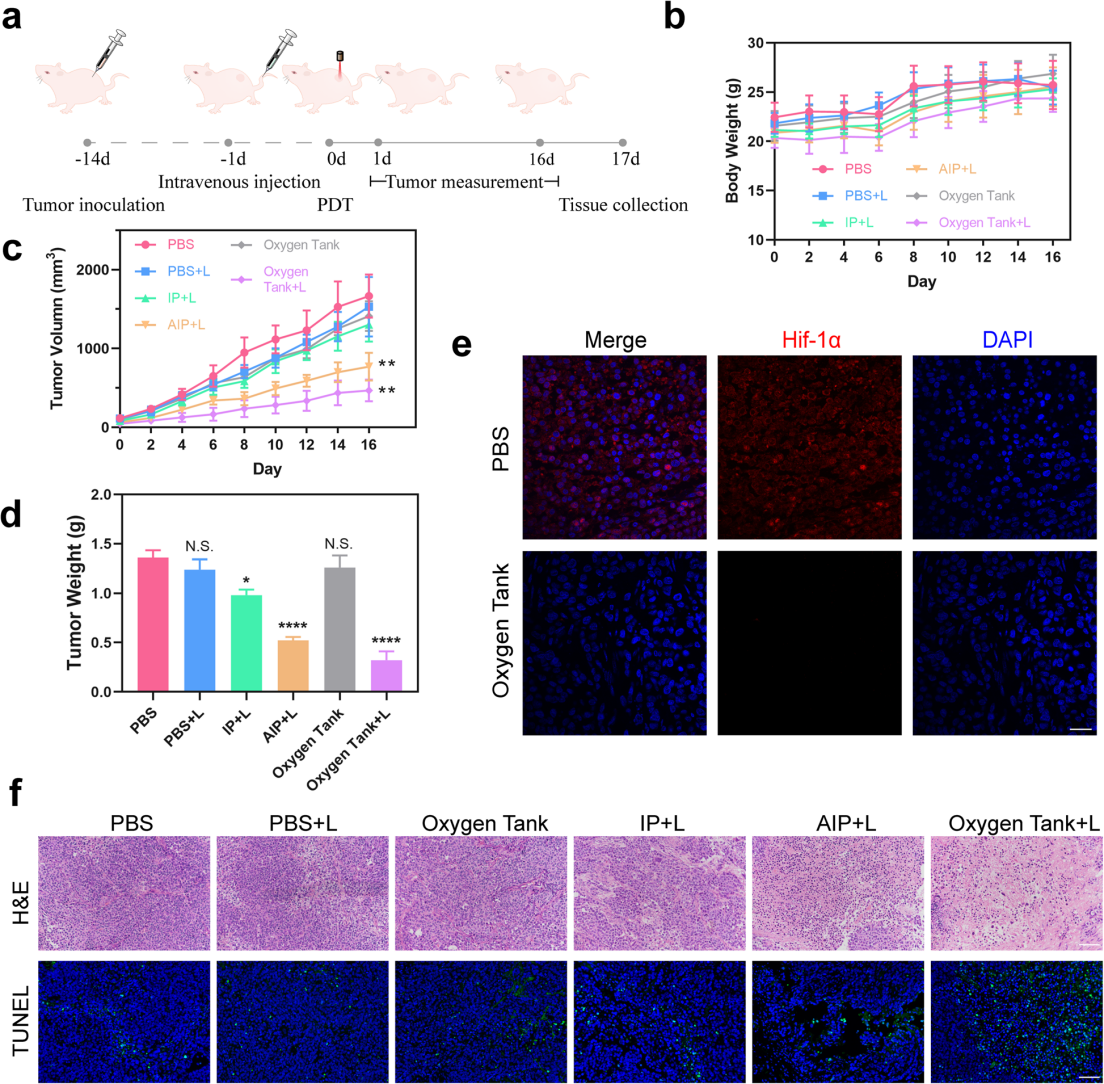
In vivo anti-tumor effect of the Oxygen Tank. **a** Schematic illustration of the PDT treatment (1 W cm − 2, 30s) after tail vein injection (200 uL, 100 μg/mL IR780, *n* = 6). **b** and **c** Body weight and tumor volume curves. **d** Weight of tumors. **e** Hif-1α staining tumor sections. The scale bar is 50 μm. **f** Photographs of the H&E and TUNEL staining of the AGS-bearing mice in different treatments (PBS, PBS with laser, Oxygen Tank, IP NPs with laser, AIP NPs with laser, and Oxygen Tank with laser). The scale bars are 100 μm. Data are shown as mean ± SD. *p < 0.05, **p < 0.01, ***p < 0.001, ****p < 0.0001, while N.S. means Not Significant

AGS tumor-bearing mice were randomly divided into six groups (PBS, PBS + laser, Oxygen tank, IP + laser, AIP + laser, and Oxygen tank + laser) to evaluate the efficacy of these NPs in designed synergistic hypoxia reversal and mt-PDT strategy (Fig. 5a). The mice of corresponding groups were irradiated with an 808 nm laser (400 mW cm^− 2^, 30 s) at 24 h post-injection. No significant change in body weight was observed among all groups, demonstrating that there was no significant acute toxicity (Fig. 5b). As shown in Fig. 5c, d, and S9a, the mice were sacrificed on day16, the tumors were excised, photographed, and weighted. These results confirmed that Oxygen tank group inhibited tumor growth most effectively. Furthermore, hematoxylin and eosin (H&E) and tdT-mediated dUTP nick-end labeling (TUNEL) results were shown in Fig. 5f. most tumor cells were severely damaged or destroyed (karyorrhexis, karyopyknosis, and karyolysis) in the Oxygen tank group. Similarly, green signals on behalf of apoptosis-positive cells in the TUNEL images exhibited similar results.

To monitor the anti-hypoxia ability of Oxygen tank in vivo, Hif-1α staining was conducted to detect whether hypoxia-related signaling pathways have been inhibited (Fig. 5e, Additional file 1: Fig. S9b, and Fig. S10). The control group exhibited a higher Hif-1α levels than IP and AIP group after 24 h post-injection. There were hardly any red signals observed in Oxygen tank group, indicating the hypoxia situation in the tumor was reversed (*p* < 0.0001). To evaluate the increased dissolved oxygen within cancer in vivo, we detected the dissolved oxygen in the gastric cancer xenografts model in vivo after different treatments (Additional file 1: Fig. S11). We utilized the oxygen probe to measure the oxygen concentration in the center of the tumor (~ 300 mm^3^). The oxygen concentration ultimately stabilized at 0.80 μmol/L and 18.13 μmol/L, respectively. AGS-bearing mice were anesthetized during the experiment.

To further test the anti-tumor effect of the Oxygen tank on a different tumor, colorectal cancer CT-26-bearing mice were also used as tumor models (Additional file 1: Fig. S12). The tumor growth curves and tumor weight analysis indicated that Oxygen tank group effectively inhibited tumor growth, while H&E slides exhibited similar results. Collectively, these results demonstrated that synergistic hypoxia reversal and mt-PDT strategy by Oxygen tank strikingly inhibited tumor growth and improved anti-tumor efficacy.

### Biosafety evaluation

**Fig. 6:**
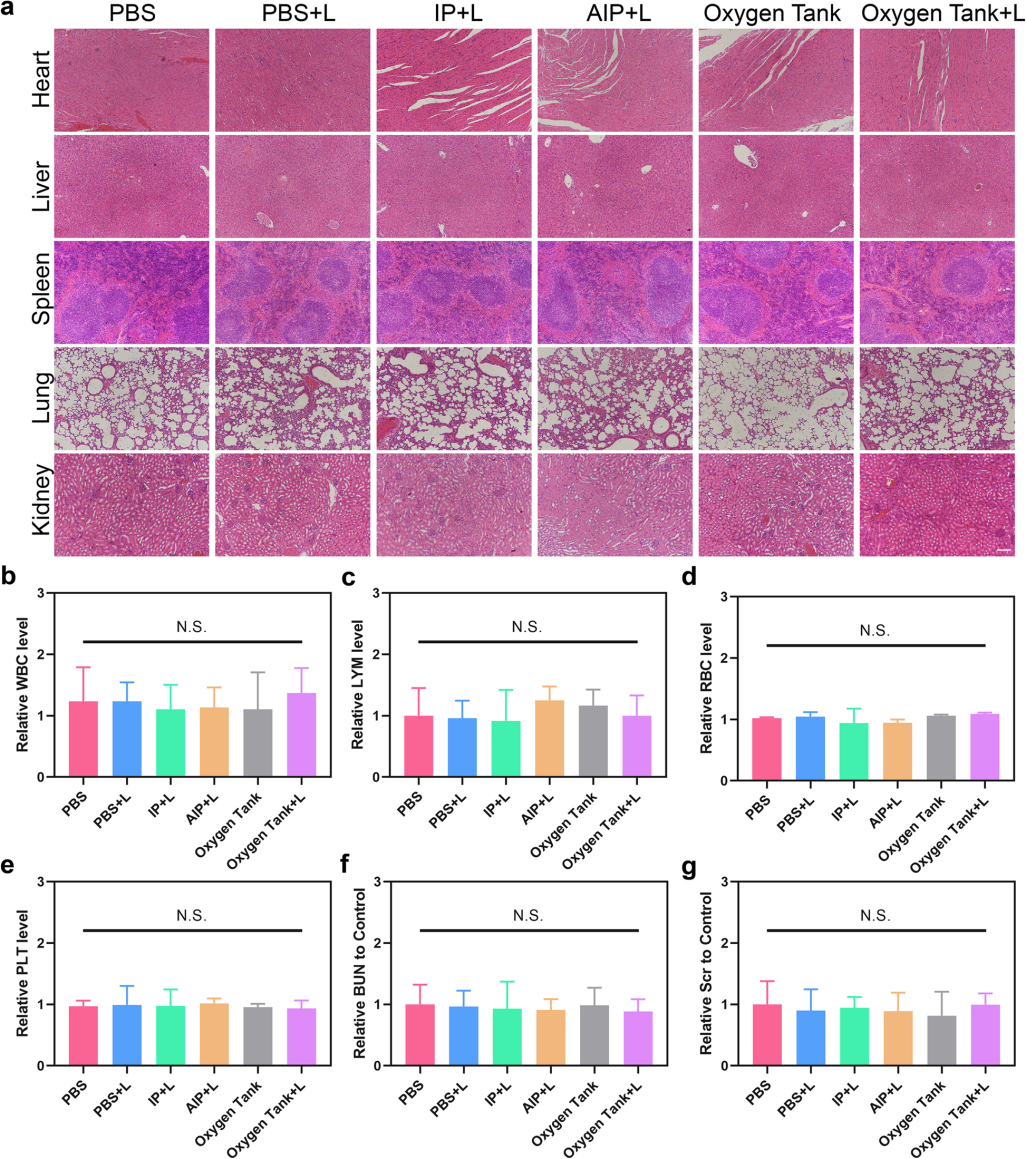
Potential long-term in vivo biosafety analysis of Oxygen Tank (200uL, 100μg/mL IR780, tail vein injection). **a** H&E staining images of major organs of the AGS-bearing mice in different treatments (PBS, PBS with laser, Oxygen Tank, IP NPs with laser, AIP NPs with laser, and Oxygen Tank with laser). The scale bar is 200 μm. **b**-**e** Hematology assay of the AGS-bearing mice in different treatments (PBS, PBS with laser, Oxygen Tank, IP NPs with laser, AIP NPs with laser, and Oxygen Tank with laser). b. WBC: white blood cell; c. LYM: lymphocytes; d. RBC: red blood cell; PLT: platelets. **f**-**g** Serum biochemical assay of the AGS-bearing mice in different treatments (PBS, PBS with laser, Oxygen Tank, IP NPs with laser, AIP NPs with laser, and Oxygen Tank with laser). **f** BUN: blood urea nitrogen; **g** Scr: serum creatinine. Data are demonstrated as mean ± SD and analyzed by the one-way ANOVA method (*n* = 3). N.S. means Not Significant

H&E staining of major organs (including heart, liver, spleen, lung, and kidney) was performed to evaluate the in vivo treatment safety (Fig. 6). Compared to the control group, there were no significant inflammation lesions, hydropic degeneration, or histopathological necrosis could be observed after treatment, indicating highly systemic biocompatibility of the Oxygen tank.

Further Hematology and serological examination were conducted to detect the potential long-term biosafety of the Oxygen tank (Fig. 6 and Additional file 1: Fig. S13). Compared to the PBS group, there was no statistical significance was observed in immune response (WBC, NEU, and LYM) and cytotoxicity (RBC and HGB) at any time point. Moreover, there was no statistical significance observed in spleen function (PLT), liver function (ALT and AST), and renal function (BUN and Scr) levels at different time points after treatments. These results demonstrated the high therapeutic biosafety of the Oxygen tank and the application potential for future clinical trials.

## Discussion

Recent progressions achieved in pre-clinical research and clinical practice improve the 5-year survival outcome of cancer patients. With the rapid development of precision medicine, researchers realized the vital role of subcellular organelles in carcinogenesis and cancer development [41, 42]. Mitochondria in cancer cells not only serve as a critical powerhouse but also drive tumor growth [43]. Some interventions based on mitochondria-targeted strategy were explored to improve anticancer efficacy, including radiotherapy [44], chemotherapy [45], and phototherapy [46]. MT-PDT we report here exhibited efficient mitochondria-targeted ability and substantial ROS generation, leading to mitochondria dysfunction and ultimately inhibition of tumor growth in different models. Notably, human tumors demonstrate high levels of glucose oxidation and tricarboxylic acid (TCA) cycle than adjacent normal tissues, displaying the necessary mitochondria metabolism pathway for tumor proliferation [47, 48]. Even in the hypoxia microenvironment, various replenishing metabolites for TCA cycle are activated and contribute to the TCA cycle hyperactivation, which fulfills the bioenergetic, biosynthetic, and redox balance requirements for tumor growth [49]. Mitochondria were proved to be required for oncogenic Kras-drive mouse models in human lung adenocarcinoma [50, 51]. Moreover, mitochondria show greater susceptibility to cancers than in normal tissues [52]. Our work suggests the essential role of the mitochondrion and its potential as an optimal selection for subcellular organelles targeted therapy.

Because mitochondria constantly consume oxygen and generate energy via OXPHOS metabolic pathway, thus, as revealed in clinical manipulation, the hypoxia microenvironment in tumors led to therapeutic resistance [53]. More importantly, radiotherapy and phototherapy consume oxygen to generate cytotoxic ROS, which leads to a more hypoxia status in subcellular organelles. Oxygen delivery by physical, chemical, or biological strategies alone could not reverse the hypoxia status. Therefore, the combination of endogenous OXPHOS inhibition and exogenous oxygen delivery can reverse the hypoxia microenvironment and improve subcellular organelle targeted therapies. Herein, a synergistic hypoxia reversal strategy was developed to enhance Mt-PDT. PFC, a commonly used artificial blood substitute, exhibits high-capacity exogenous oxygen supplied and is reported to successfully enhance the PDT effect [18, 23].

ATO is the mitochondria complex III inhibitor, which effectively inhibits mitochondrial respiration to economize endogenous oxygen consumption [54, 55]. Notably, complex I inhibitor (e.g., metformin) could not increase ROS generation because the electron released from complex I direct to the mitochondrial matrix, and quickly becomes inactive due to matrix-antioxidant enzyme systems (e.g., NADH) [56]. Constantly, complex III inhibitors ATO increases ROS production in the cytoplasm because ROS generation from the Qi site is directed to intermembrane space and away from matrix-antioxidant enzyme systems, thus increasing ROS generation and not affected by antioxidant defense [57–60].

Endogenous oxygen consumption inhibition and exogenous oxygen delivery are quite promising for synergistic oxygen-consumed interventions, such as radiotherapy and phototherapy. Here we combined the synergistic hypoxia reversal and organelle targeted therapy, and eventually obtained substantial ROS generation and a powerful anti-cancer effect. Notably, oxygen supply and subcellular targeted ROS generation increase the therapeutic sensitivity to various interventions [61–63]. ROS-mediated immunogenic cell death may boost an antitumor immune response and amplify immunotherapy [64, 65]. Oxidized mitochondrial DNA triggered by ROS activates STING signaling and promotes anti-tumor effect [66–68]. Additionally, mitochondrial DNA stress leads to autophagy-dependent ferroptosis [6]. These findings broaden the window for future application of oxygen amplifying subcellular targeted therapy.

Additionally, all the components from the Oxygen tank are bio-compatible. PFC is widely used as artificial blood and ATO is utilized to treat malaria. They are all approved by FDA. Loaded by the artificial RBC, we displayed that lipophilic drugs effectively prolong blood circulation time and promote tumor permeation [18, 19, 69]. Safety concerns always impede the clinical translation of multiple nanomedicines, while FDA-approved drugs loaded by bio-mimic systems may fill the gap between research and application [70]. On the whole, our strategy displaying high safety and efficacy for tumor eradication is worth looking forward to clinical translation.

## Conclusion

In summary, we have successfully fabricated a novel RBC-mimic system, RBC@AIP artificial blood cells, to combat hypoxia in the tumor microenvironment and further exhibited anti-tumor activity, in which the PFC core could dissolve a large quantity of oxygen while the RBCm coating could prolong blood circulation. Importantly, the introduction of FDA-approved ATO, which inhibits mitochondrial respiration, greatly decreases endogenous oxygen consumption. After intravenous injection, the Oxygen tank could deliver oxygen into the tumor and inhibit tumor mitochondrial respiration simultaneously, thus dramatically enhancing the overall oxygenation status. Subsequently, due to the synergistic tumor hypoxia reversal, amplified IR780 induced Mt-PDT remarkably inhibited tumor growth. This exogenous oxygen supply and endogenous oxygen economization synergistic strategy based on artificial red blood cells were first developed and applied in Mt-PDT. Compared to our previously reported human serum albumin (HSA)-coated PFC for enhanced PDT [19, 35], this current technique focused on tumor inherent oxygen metabolism and bioactivity of nanoparticles, and thoroughly reversed tumor hypoxia. Additionally, all components are clinical approved or highly biocompatible, such Oxygen tank NPs as artificial red blood cells would serve as a simple, safe, and effective oxygen modulator to enhance tumor oxygenation. Furthermore, such a strategy would also exert the potential to improve outcomes of various therapies such as radiotherapy and immunotherapy, in which tumor hypoxia remains a factor for therapeutic resistance.

## Experimental section

### Materials

ATO, PFC (Perfluorotributylamine), and IR780 were purchased from Sigma Aldrich (Missouri, USA). DSPE-PEG2000, DSPE- PEG2000-COOH, and cholesterol were purchased from Shanghai Yuanye Bio-Technology (Shanghai, China). Lecithin (from eggs) was purchased from MedChemExpress (Shanghai, China).

### Synthesis of AIP NPs

Briefly, DSPE-PEG2000, DSPE- PEG2000-COOH, lecithin, cholesterol, ATO, and IR780 were dissolved in 5 ml dichloromethane. Next, the dichloromethane was removed by rotary evaporation and formed lipid films. Then 2.8 ml of pure water was added gradually. The film was peeled off by 10 min sonication. 1.2 ml PFTBA was added under high-speed dispersion in an ice bath to form AIP NPs. IR780-PFTBA (IP) NPs can be formed without ATO, while AIP NPs can be formed by the addition of oil instead of PFTBA.

### Synthesis of oxygen tank NPs

The RBCm-derived vesicles were collected according to a previous study [2]. In brief, to remove the plasma and other cells, freshly heparinized blood was centrifuged at 3500 rpm for 10 min. Then, collected RBCs were washed by 1 × PBS 3 times. To remove intracellular contents, washed RBCs were resuspended in 0.25 × PBS in an ice bath for 2 h by hypotonic treatment. After 9000 rpm centrifugation for 15 min and washed 3 times, the RBC ghosts were collected. Next, the RBC ghosts were sonicated for 2 min in an ice bath, and subsequently extruded through the 400 nm polycarbonate porous membrane (Millipore). Obtained RBCm-derived vesicles were stored at − 20 °C.

The Oxygen tank NPs were fabricated by AIP and RBCm. One ml AIP was mixed with RBCm derived vesicles prepared from 2 ml whole blood and extruded 10 times through a 400 nm polycarbonate porous membrane. The resulting Oxygen tank was collected after centrifugation to remove excess materials remaining in the supernatant.

Moreover, sodium dodecyl sulfate-polyacrylamide gel electrophoresis (SDS-PAGE) was used to detect the distribution of characteristic protein bands of RBCm and Oxygen tank. Samples were mixed with SDS sample buffer and then boiled for 5 min at 100 °C to denature the protein. Next, samples were run on a 4–20% Tris-Gly minigel in a running buffer by BIO-RAD Electrophoresis System at 80 V for 0.5 h and then at 120 V for 1 h to separate protein brands. The resulting polyacrylamide gel was stained by Coomassie brilliant blue for 1 h and washed off excess blue by decolorizing for visualization of proteins.

### Characterization

The morphology and size of AIP NPs and Oxygen tank NPs were detected by Transmission electron microscopy (TEM, JEOL, Japan). The mean particle diagram and zeta potential were measured by dynamic light scattering (DLS, 90Plus, Brookhaven Instrum. Corp). UV-vis (UV-2450, Shimadzu, Japan) was used to determine the absorption spectrum in different samples. The PBS results of AIP NPs and Oxygen tank NPs in PBS were monitored every 6 h to identify their stability. To confirm the encapsulation of ATO in the Oxygen tank, with mobile phase acetonitrile-phosphate buffer, 0.01 M, pH 7.0 (90,10, v/v), and UV detection at 254 nm [71, 72]. Chromatographic separation was carried out using a C_18_ column with a flow rate of 1.0 ml/min. To confirm the encapsulation of PFC in the Oxygen tank, we added ethanol into Oxygen Tank. After centrifugation, PFC was separated from the ethanol solution.

### Cells culture and animal model

The human gastric cancer cell line AGS was purchased from the American Type Culture Collection (Manassas, VA, USA). The mouse CT26 colorectal cancer cells were purchased from China Type Culture Collection. The cell lines were cultured in RPMI 1640 medium (Gibco, Waltham, MA, USA) as supplemented with 10% fetal bovine serum (FBS; Gibco, Waltham, MA, USA) and 1% penicillin/streptomycin (Gibco, Waltham, MA, USA) at 37 °C in 5% CO_2_. Cell construction and experiment were conducted when the cells reached 80% confluence.

All animal tests and experimental procedures used in this experiment were performed following protocols approved by the Institutional Animal Care and Use Committee of Nanjing University (NJU-IACUC, IACUC2003160). To establish the gastric cancer xenografts model, AGS cells were suspended into PBS (1 × 10^7^ cells in 100 μl each mouse) and then subcutaneously injected into the right upper extremity area of nude mice. The volume of tumor tissues was calculated as [length×(width)^2^/2]. CT26 colorectal cancer model was established by a similar method using Balb/C mice.

### Detection of hypoxia in cancer cells

To demonstrate the synergistic hypoxia relief ability of AIO, AIP, and Oxygen tank, an oxygen probe (OX-NP, 1.6 × 40 mm-needle sensor for piercing, Unisense A/S CO.LTD) was used to measure the oxygen concentration of culture media of different groups. To further measure the hypoxia reverse ability of the Oxygen tank, AGS cells were seeded into confocal wells (20mm-glass -bottom dishes, NEST Biotechnology Co. Ltd.) at a density of 2.5 × 10^4^ cells per well. After 24 h incubation, corresponding wells were placed in the hypoxia incubator (10% O_2_) to simulate a hypoxia condition. After being washed 3 times, the cells were evaluated by hypoxia-inducible factor-1α.

### Subcellular localization of oxygen tank

To verify the mitochondrial targeting of IR780, lysosomes, and mitochondria were labeled, respectively [3]. After incubation with Oxygen tank NPs for 4 h, AGS cells were incubated with LysoTracker green DND-26 for another 30 min to label lysosomes, or MitoTracker Green FM for another 60 min to label mitochondria. The cells were then washed and monitored by confocal microscope (OLYMPUS FV3000) to obtain the fluorescent images. The Pearson correlation (PC) coefficient and colocalization analysis results were calculated using ImageJ software.

### Detection of ROS generation

Six groups were divided (PBS, PBS + L, Oxygen tank, IP + L, AIP + L, and Oxygen tank+L) to measure the ROS generation by H_2_DCFDA (a ROS sensitive probe). After incubation with Oxygen tank NPs for 4 h, AGS cells were incubated with H_2_DCFDA and protected from light. After 808 nm laser irradiation, the fluorescence images were measure by a confocal microscope. The quantificational analysis was conducted using ImageJ software.

### Measurement of mitochondrial function

The activity of mitochondria was monitored by mitochondrial membrane potential using the JC-1 assay kit (Beyotime Biotechnology, Shanghai, China). Firstly, AGS cells were cultured with PBS, IP NPs, ATO, and Oxygen tank for 4 h. then, the JC-1 assay kit was added to AGS cells for 20 min. Then the cells were washed 3 times. Normally polarized mitochondria (λex/λem = 525 nm/590 nm) and abnormally depolarized mitochondria (λex/λem = 490 nm/530 nm) were detected by confocal microscope.

### In vitro anti-tumor efficacy

After identifying the ROS generation and mitochondria targeting of the Oxygen tank, Cell Counting kit-8 (CCK-8) and Calcein-AM/Propidium Iodide were used to detect the anti-tumor effect in vitro. Cancer cells were cultured in wells for 24 h and co-incubated with different NPs for another 4 h. After that, the cells were washed twice, and then particular groups were exposed to 808 nm laser (1 W/cm^2^, 30 s). Then, 10 μl CCK-8 working solution was added to each well for all groups and followed by further co-incubation. The absorbance value at 450 nm of every well was detected by the microplate reader according to the manufacturer. As for Calcein-AM/Propidium Iodide double stain detection, 100 μl Calcein-AM/Propidium Iodide double stain working solution was added into all groups according to the manufacturer’s instructions. After 30 min co-incubation, cells were washed 3 times and monitored by a confocal microscope. The living cells were stained with Calcein-AM while dead cells were stained with Propidium Iodide. The quantificational analysis was conducted using ImageJ software.

### In vivo biodistribution and NIR fluorescence imaging

Subcutaneous tumor-bearing nude mice were injected with the Oxygen tank (200 μl, 100 μg/mL IR780, tail vein injection). NIR fluorescence images were acquired by the CRI maestro system (λex/λem = 740/810 nm) at different time points (1, 3, 5, 8, 12, 24, and 48 h). At the end of the experiment, the mice were euthanized while tumors and organs (heart, spleen, liver, lung, and kidney) were collected for fluorescence imaging. Fluorescence signals were calculated for semi-quantitative biodistribution analysis.

### In vivo PDT evaluation

PDT treatment was performed on the 14th day after inoculation of tumor cells. The testing groups were as follow: group 1: saline; group 2: saline + laser; group 3: IP NPs + laser; group 4: AIP NPs + laser; group 5: Oxygen tank NPs; group 6: Oxygen tank NPs + laser (*n* = 6). Different NPs were injected (200 μl, IR780 100 μg/mL) in corresponding groups. Tumor volume and body weight of mice were monitored every 2 days. After 14 days, the mice were sacrificed and tumor tissues were collected. Finally, collected tumor tissues were stained with H&E (hematoxylin and eosin), TUNEL (tdT-mediated dUTP nick-end labeling), and HIF-1α for further histopathology analysis.

### Safety analysis

To evaluate the biocompatibility and toxicity of the Oxygen tank, male AGS-bearing mice were anesthetized using a 1.5% pentobarbital sodium solution. The mice’s blood was collected via extirpating with the naked eye for biochemical assays. Once collected, the blood was drawn in a pro-coagulation tube and centrifuged at 3000 rpm for 15 min to separate serum. The serological liver function was detected by alanine aminotransferase (ALT) and aspartate aminotransferase (AST) level, while the renal function was studied by blood urea nitrogen (BUN) and serum creatinine (Scr). Major organs were collected for H&E staining on different day post intravenous injection of the Oxygen tank.

### Statistical analysis

The Graphpad Prism (version 8.01) software were performed to identify statistical analyses with a significance level of *p < 0.05, **p < 0.01, ***p < 0.001, and ****p < 0.0001. Data were presented as mean ± standard deviation (SD).

## Supplementary Information

**Additional file 1: Fig. S1**. Characterization of Oxygen Tank. a) Hydrodynamic diameters of Oxygen Tank NPs. Inset shows photographs and TEM images of these NPs. b) In vitro safety analysis of Oxygen Tank. c) Standard curves of Oxygen Tank NPs. d) Stability of Oxygen Tank and AIP within 96 h. Data are demonstrated as mean ± SD (*n* = 3). **Fig. S2** Chromatogram of ATO. Before analysis, ATO was dissolved in methanol (final concentration 50 μg/mL). For HPLC conditions see Experimental Section. The figures beside the peaks are retention times in minutes and responses in mAU. **Fig. S3** Chromatogram of Oxygen Tank. Before analysis, emulsion breaking was performed using methanol. Ultimately the Oxygen Tank was diluted for 10 folds. For HPLC conditions see Experimental Section. The figures beside the peaks are retention times in minutes and responses in mAU. **Fig. S4** Identification of PFC in Oxygen Tank. a) before centrifugation. b) after centrifugation. **Fig. S5.** Quantitative result of confocal fluorescence images of Hif-1α staining of AGS cells after different treatments (PBS in hypoxia condition, IP NPs in hypoxia condition, AIP NPs in hypoxia condition, Oxygen Tank in hypoxia condition, and PBS in normoxia condition). Data are showed as mean ± SD (*n* = 3). **Fig. S6**. The cellular uptake in AGS and CT26 cells. The flow cytometry of AGS (a) and CT26 cells treated with AIP and Oxygen Tank (IR780, 4 μg/mL) for 0, 1, 2, and 4 h. Data are demonstrated as mean ± SD (*n* = 3). c) The fluorescence images of CT26 tumor bearing mice at different times. Oxygen Tank exhibited an enhanced accumulation in tumor (200 μL, 100 μg/mL IR780). **Fig. S7**. Proportion of green cells in total cells from CLSM images of AGS cells determined by CAM/PI double stain kit (*n* = 3). Data are showed as mean ± SD. *p < 0.05. **Fig. S8**. Biodistribution of Oxygen Tank (200uL, 100μg/mL IR780, tail vein injection). a) The in vivo fluorescence images of AGS bearing mice at different time points (*n* = 6). b) Ex vivo NIR images of major organs and tumors at 24 h post intravenous injection (*n* = 4). c) Quantification of the in vivo fluorescence signal intensity of tumor area after injection of Oxygen Tank (*n* = 6, S8a). d) Quantification of the in vivo fluorescence signal intensity of Oxygen Tank in different organs at 24 h post intravenous injection (*n* = 4, S8b). Data are demonstrated as mean ± SD (*n* = 4). **Fig. S8**. Biodistribution of Oxygen Tank (200uL, 100μg/mL IR780, tail vein injection). a) The in vivo fluorescence images of AGS bearing mice at different time points (*n* = 6). b) Ex vivo NIR images of major organs and tumors at 24 h post intravenous injection (*n* = 4). c) Quantification of the in vivo fluorescence signal intensity of tumor area after injection of Oxygen Tank (*n* = 6, S8a). d) Quantification of the in vivo fluorescence signal intensity of Oxygen Tank in different organs at 24 h post intravenous injection (*n* = 4, S8b). Data are demonstrated as mean ± SD (*n* = 4). **Fig. S9.** a) Photograph of tumors of in vivo anti-tumor evaluation (*n* = 5). b) Hif-1α staining tumor sections. The scale bar is 50 μm. **Fig. S10.** Quantitative result of Hif-1α staining tumor sections after different treatments (PBS, IP NPs, AIP NPs, and Oxygen Tank). Data are showed as mean ± SD (*n* = 3). **Fig. S11.** The dissolved oxygen curves in tumor site after different treatments (PBS or Oxygen Tank, 200uL, 100μg/mL IR780, tail vein injection). Start recording once the oxygen probe was inserted into the tumor in vivo. AGS bearing mice were anesthetized during the experiment. **Fig. S12**. In vivo anti-tumor effect of Oxygen Tank in CT26 bearing mice (200 μL, 100 μg/mL, tail vein injection, *n* = 6). a) The body weight curves. b) The tumor volume curves. c) Weight of tumors. d) H&E staining tumor sections. The scale bar is 200um. Data are showed as mean ± SD. *p < 0.05, while N.S. means Not Significant. **Fig. S13**. Potential long-term in vivo biosafety analysis of Oxygen Tank (200 μL, 100 μg/mL IR780, tail vein injection). a-c) Hematology assay (NEU: neutrophils; HGB: hemoglobin; PLT: platelets). d-e) Serum biochemical assay (ALT: alanine aminotransferase; AST, aspartate aminotransferase). Data are demonstrated as mean ± SD and analyzed by one-way ANOVA method (n = 3). N.S. means Not Significant. **Table S1.** Peak table of high-performance liquid chromatography result of ATO. **Table S2.** Peak table of high-performance liquid chromatography results of Oxygen Tank.

## Data Availability

We declared that materials described in the manuscript, including all relevant raw data, will be freely available to any scientist wishing to use them for non-commercial purposes. The datasets used and analyzed during the current study are available from the corresponding author upon reasonable request without breaching participant confidentiality.

## Abbreviations

PDT: photodynamic therapy
ATO: atovaquone
PFC: perfluorocarbon
RBCm: red blood cell membrane
ROS: reactive oxygen species
OXPHOS: oxidative phosphorylation
ETC: electro-transport chain
HIF-1α: hypoxia-inducible factor-1α
WBC: white blood cell
LYM: lymphocytes
RBC: red blood cell
PLT: platelets
BUN: blood urea nitrogen
Scr: serum creatinine
