# Supplementary material for: Oxygen tank for synergistic hypoxia relief to enhance mitochondria-targeted photodynamic therapy
Source: Biomater Res. 2022 Sep 22;26:47. doi: 10.1186/s40824-022-00296-0 (PMC9502906; doi:10.1186/s40824-022-00296-0)
Supplement: Supplementary file 1 — Additional file 1: Fig. S1. Characterization of Oxygen Tank. a) Hydrodynamic diameters of Oxygen Tank NPs. Inset shows photographs and TEM images of these NPs. b) In vitro safety analysis of Oxygen Tank. c) Standard curves of Oxygen Tank NPs. d) Stability of Oxygen Tank and AIP within 96 h. Data are demonstrated as mean ± SD (n = 3). Fig. S2 Chromatogram of ATO. Before analysis, ATO was dissolved in methanol (final concentration 50 μg/mL). For HPLC conditions see Experimental Section. The figures beside the peaks are retention times in minutes and responses in mAU. Fig. S3 Chromatogram of Oxygen Tank. Before analysis, emulsion breaking was performed using methanol. Ultimately the Oxygen Tank was diluted for 10 folds. For HPLC conditions see Experimental Section. The figures beside the peaks are retention times in minutes and responses in mAU. Fig. S4 Identification of PFC in Oxygen Tank. a) before centrifugation. b) after centrifugation. Fig. S5. Quantitative result of confocal fluorescence images of Hif-1α staining of AGS cells after different treatments (PBS in hypoxia condition, IP NPs in hypoxia condition, AIP NPs in hypoxia condition, Oxygen Tank in hypoxia condition, and PBS in normoxia condition). Data are showed as mean ± SD (n = 3). Fig. S6. The cellular uptake in AGS and CT26 cells. The flow cytometry of AGS (a) and CT26 cells treated with AIP and Oxygen Tank (IR780, 4 μg/mL) for 0, 1, 2, and 4 h. Data are demonstrated as mean ± SD (n = 3). c) The fluorescence images of CT26 tumor bearing mice at different times. Oxygen Tank exhibited an enhanced accumulation in tumor (200 μL, 100 μg/mL IR780). Fig. S7. Proportion of green cells in total cells from CLSM images of AGS cells determined by CAM/PI double stain kit (n = 3). Data are showed as mean ± SD. *p < 0.05. Fig. S8. Biodistribution of Oxygen Tank (200uL, 100μg/mL IR780, tail vein injection). a) The in vivo fluorescence images of AGS bearing mice at different time points (n = 6). b) Ex vivo NIR images of [file 40824_2022_296_MOESM1_ESM.docx]

Supporting Materials for

# Oxygen Tank for synergistic hypoxia relief to enhance mitochondria-targeted photodynamic therapy

Xianghui Li, Haoran Wang, Zhiyan Li, Dandan Li, Xiaofeng Lu, Shichao Ai, Yuxiang Dong, Song Liu^*^, Jinhui Wu^*^, Wenxian Guan^*^

^*^Corresponding authors. Email: liusong@njglyy.com, wuj@nju.edu.cn, medguanwx@163.com


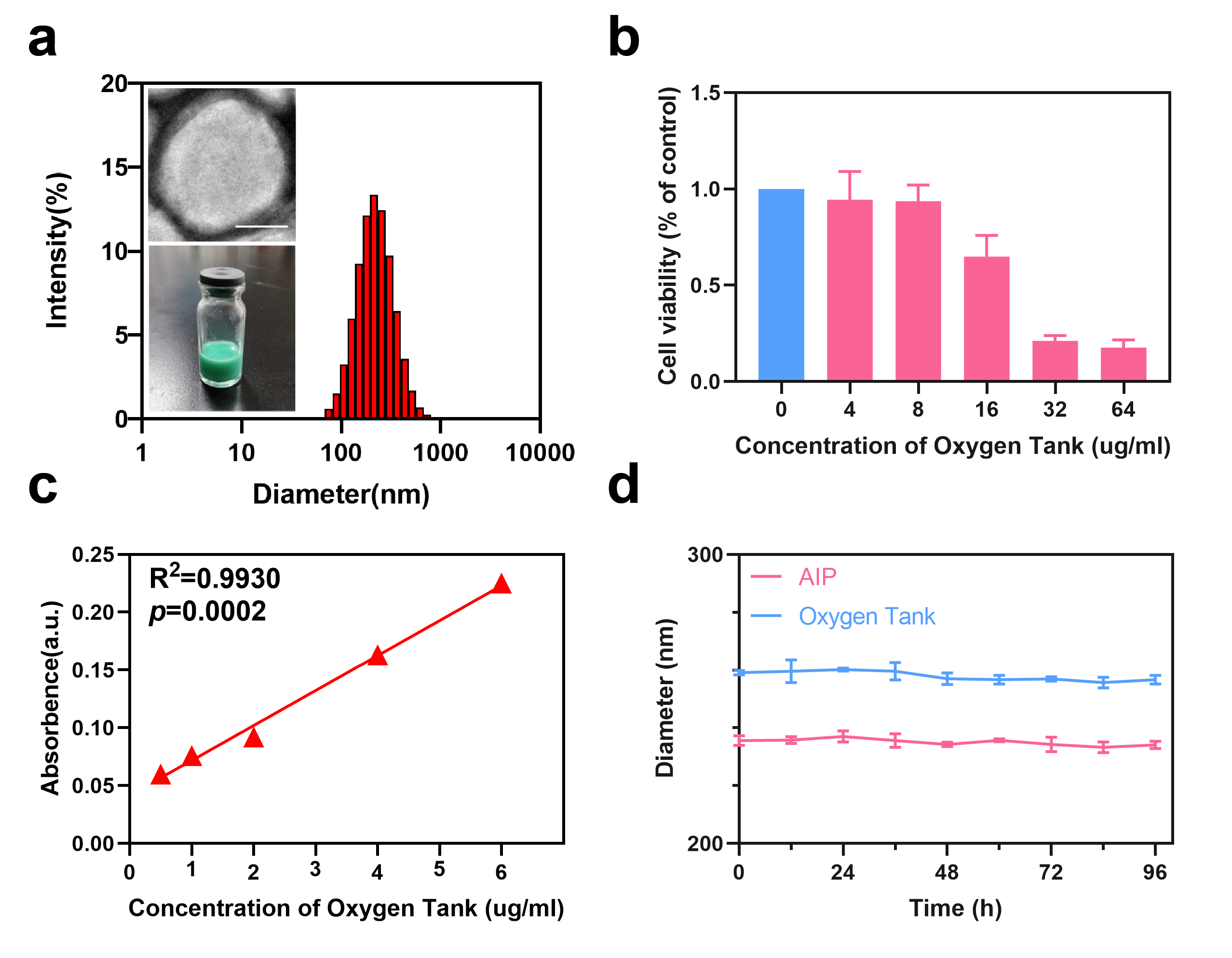


***Figure S1****. Characterization of Oxygen Tank. a) Hydrodynamic diameters of Oxygen Tank NPs. Inset shows photographs and TEM images of these NPs. b) In vitro safety analysis of Oxygen Tank. c) Standard curves of Oxygen Tank NPs. d) Stability of Oxygen Tank and AIP within 96 h.* *Data are demonstrated as mean ± SD (n=3).*


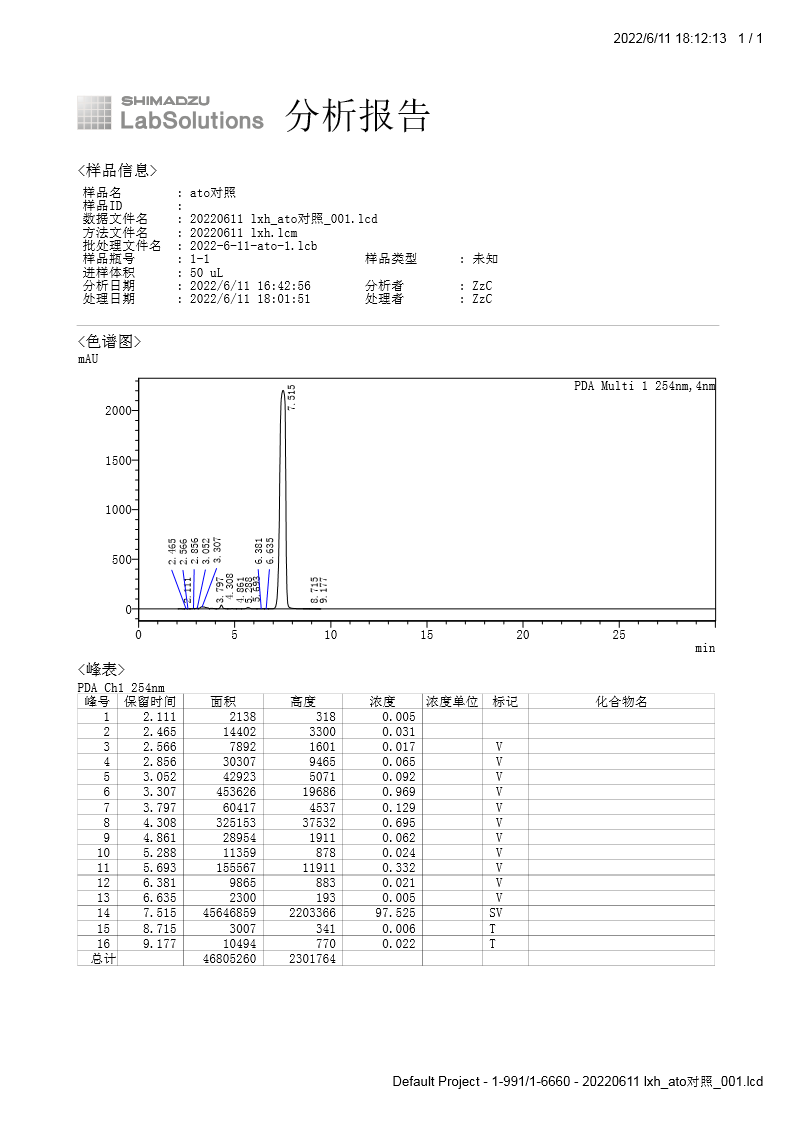


***Figure S2 Chromatogram of ATO.*** *Before analysis, ATO was dissolved in methanol (final concentration 50 μg/mL). For HPLC conditions see Experimental Section. The figures beside the peaks are retention times in minutes and responses in mAU.*


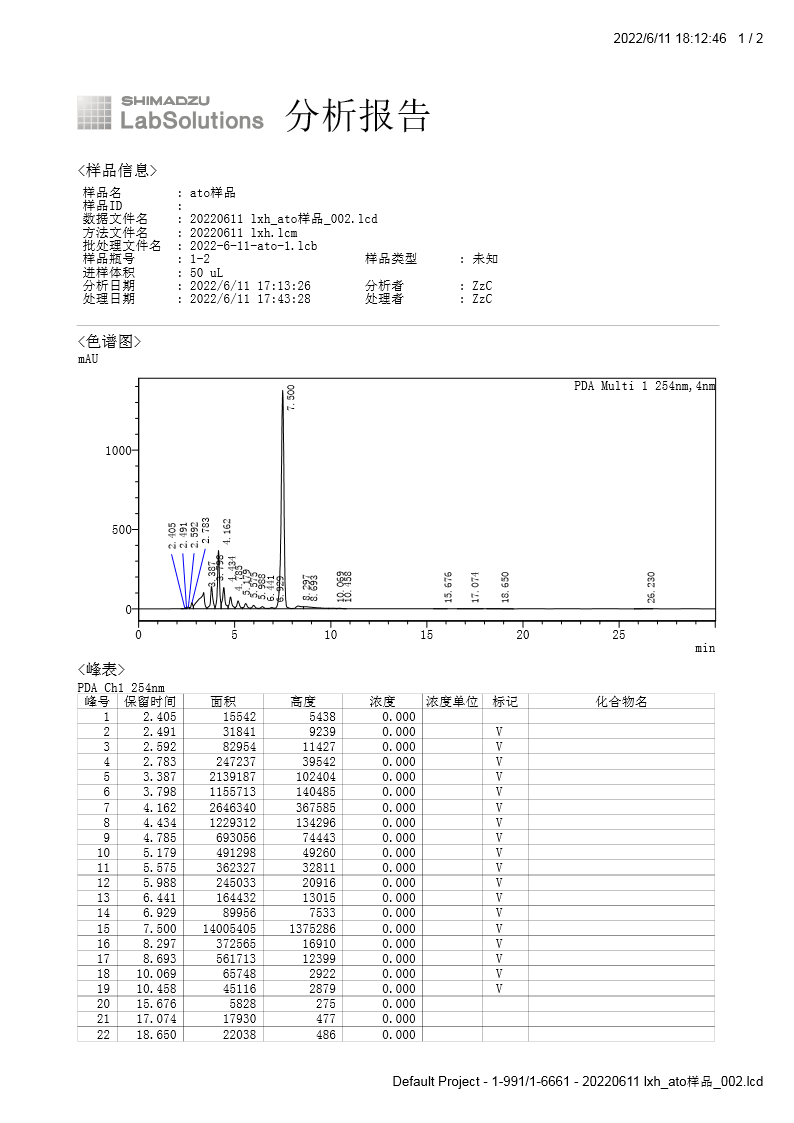


***Figure S3 Chromatogram of Oxygen Tank.*** *Before analysis, emulsion breaking was performed using methanol. Ultimately the Oxygen Tank was diluted for 10 folds. For HPLC conditions see Experimental Section. The figures beside the peaks are retention times in minutes and responses in mAU.*


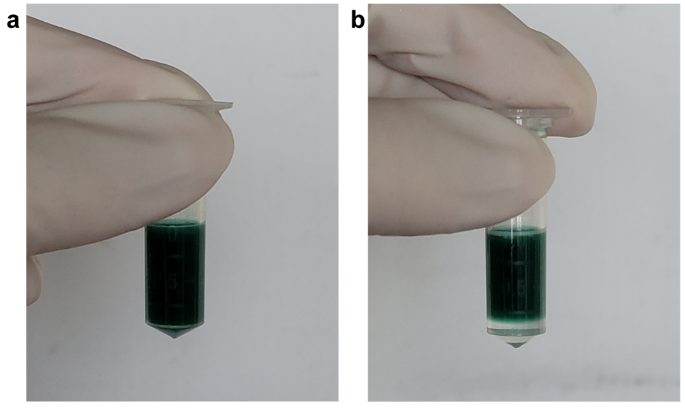


***Figure S4 Identification of PFC in Oxygen Tank.*** *a）before centrifugation. b) after centrifugation.*


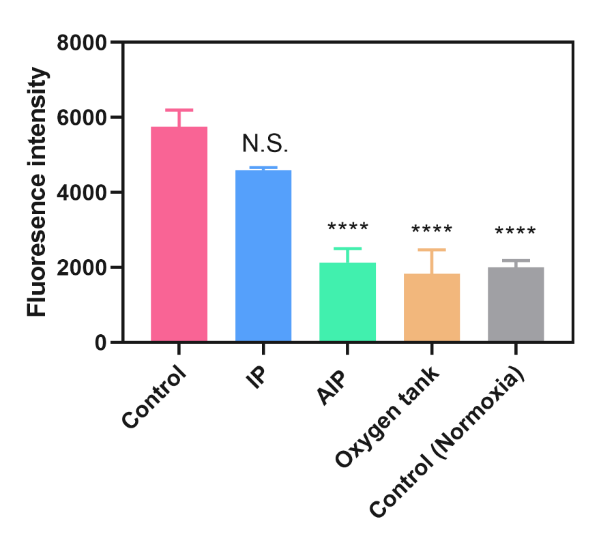


***Figure S5.*** *Quantitative result of confocal fluorescence images of Hif-1α staining of AGS cells after different treatments (PBS in hypoxia condition, IP NPs in hypoxia condition, AIP NPs in hypoxia condition, Oxygen Tank in hypoxia condition, and PBS in normoxia condition). Data are showed as mean ± SD (n=3).*


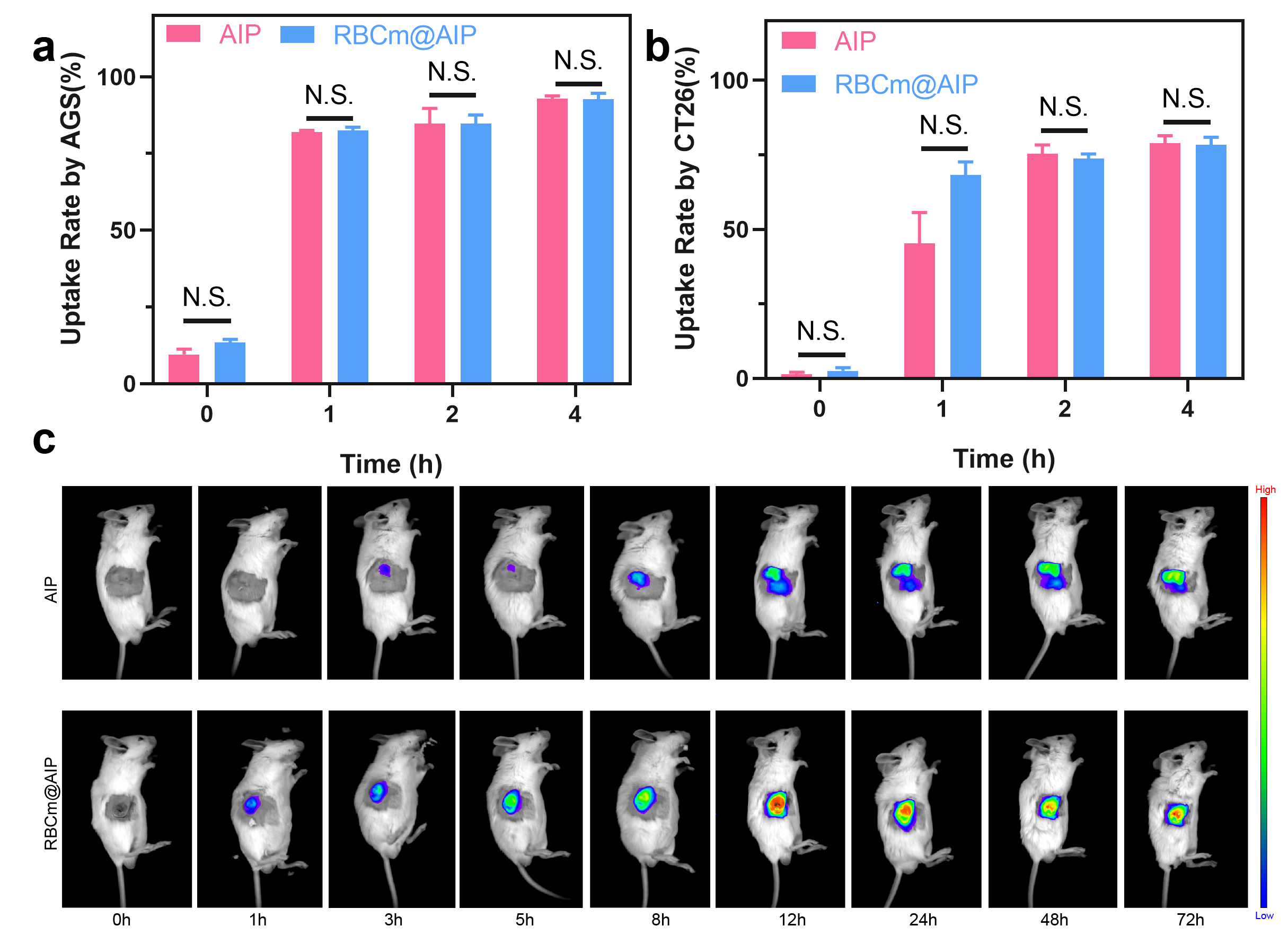


***Figure S6****. The cellular uptake in AGS and CT26 cells. The flow cytometry of AGS (a) and CT26 cells treated with AIP and Oxygen Tank (IR780, 4 μg/mL) for 0, 1, 2, and 4 h. Data are demonstrated as mean ± SD (n=3). c) The fluorescence images of CT26 tumor bearing mice at different times. Oxygen Tank exhibited an enhanced accumulation in tumor (200 μL, 100 μg/mL IR780).*

**
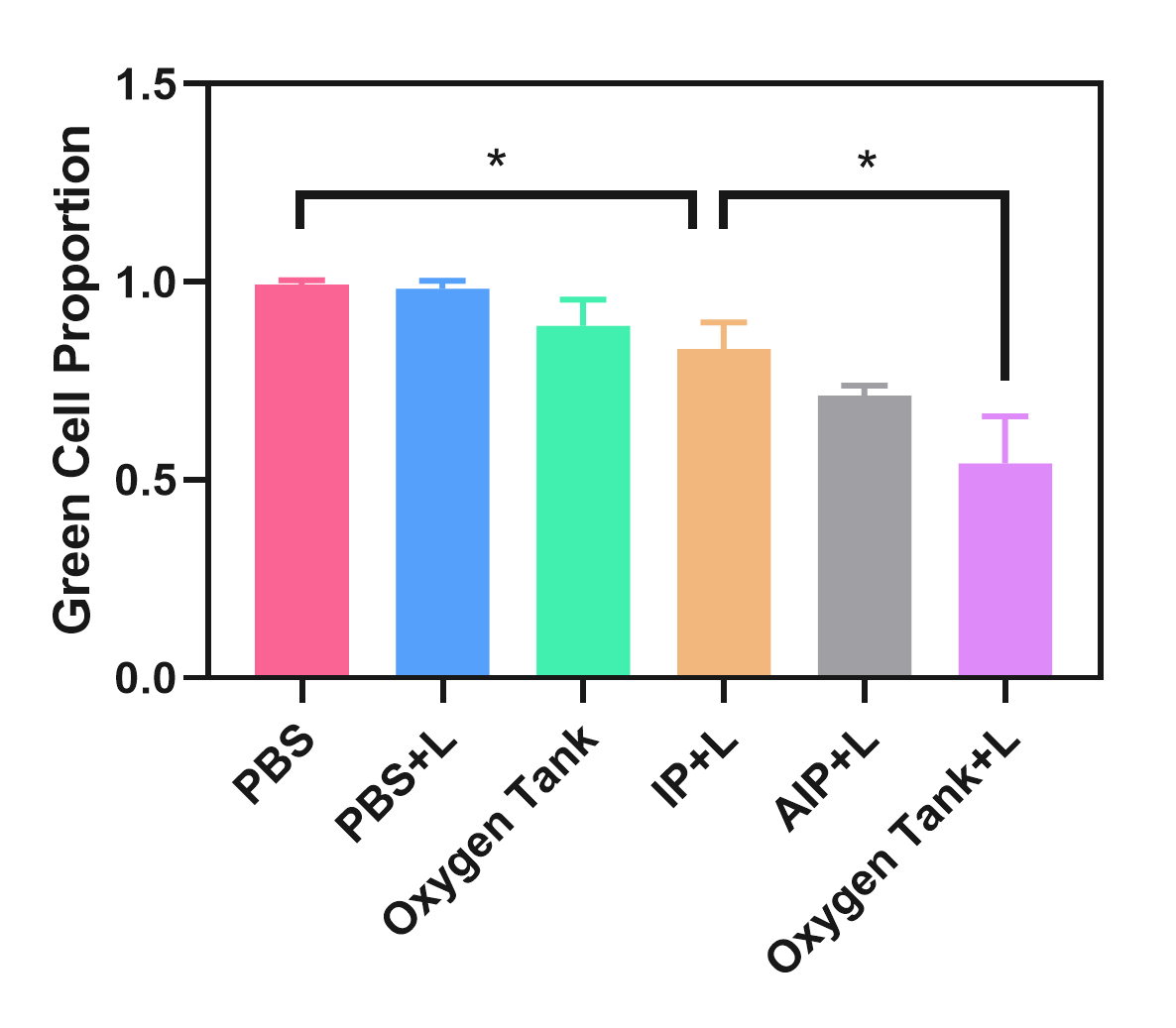
**

***Figure S7****. Proportion of green cells in total cells from CLSM images of AGS cells determined by CAM/PI double stain kit (n=3).* *Data are showed as mean ± SD. *p<0.05.*

**
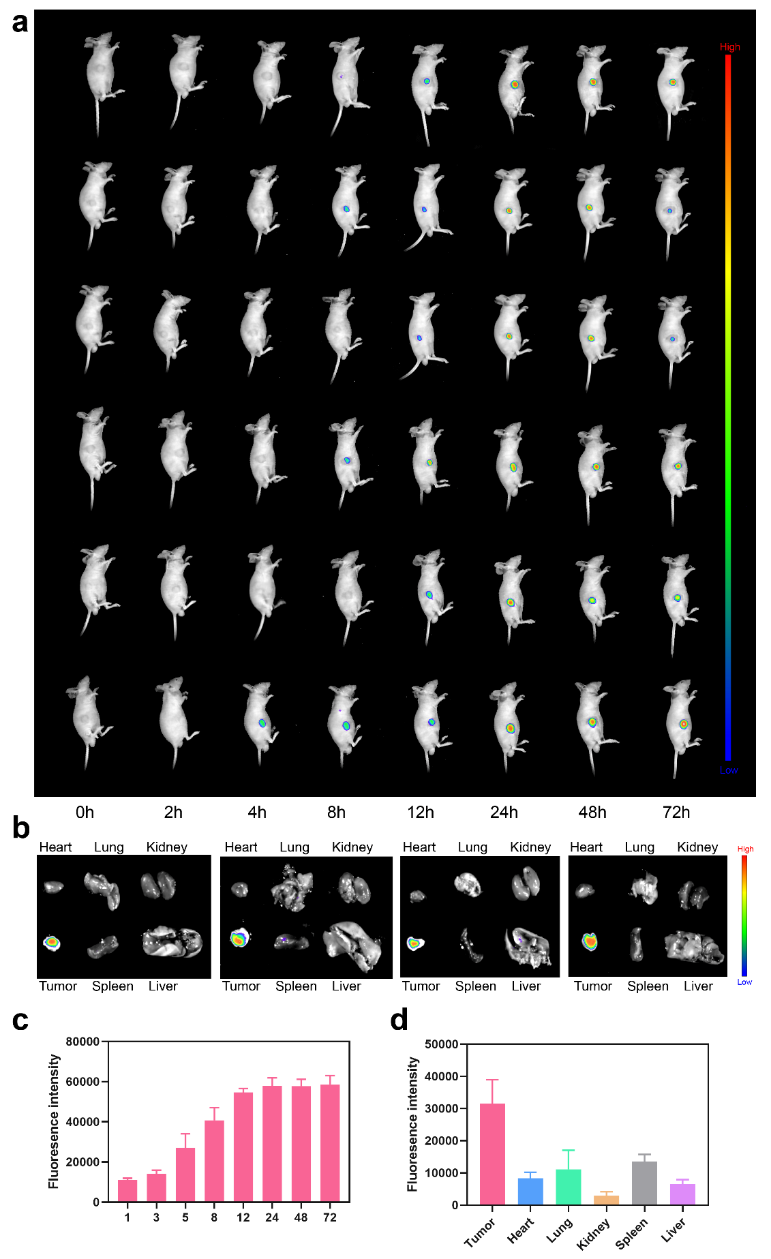
**

­­***Figure S8****. Biodistribution of Oxygen Tank (**200uL, 100ug/mL IR780, tail vein injection). a) The in vivo fluorescence images of AGS bearing mice at different time points (n=6). b) Ex vivo NIR images of major organs and tumors at 24 h post intravenous injection (n=4). c) Quantification of the in vivo fluorescence signal intensity of tumor area after injection of Oxygen Tank (n=6, S8a). d) Quantification of the in vivo fluorescence signal intensity of Oxygen Tank in different organs at 24 h post intravenous injection (n=4, S8b). Data are demonstrated as mean ± SD (n=4).*
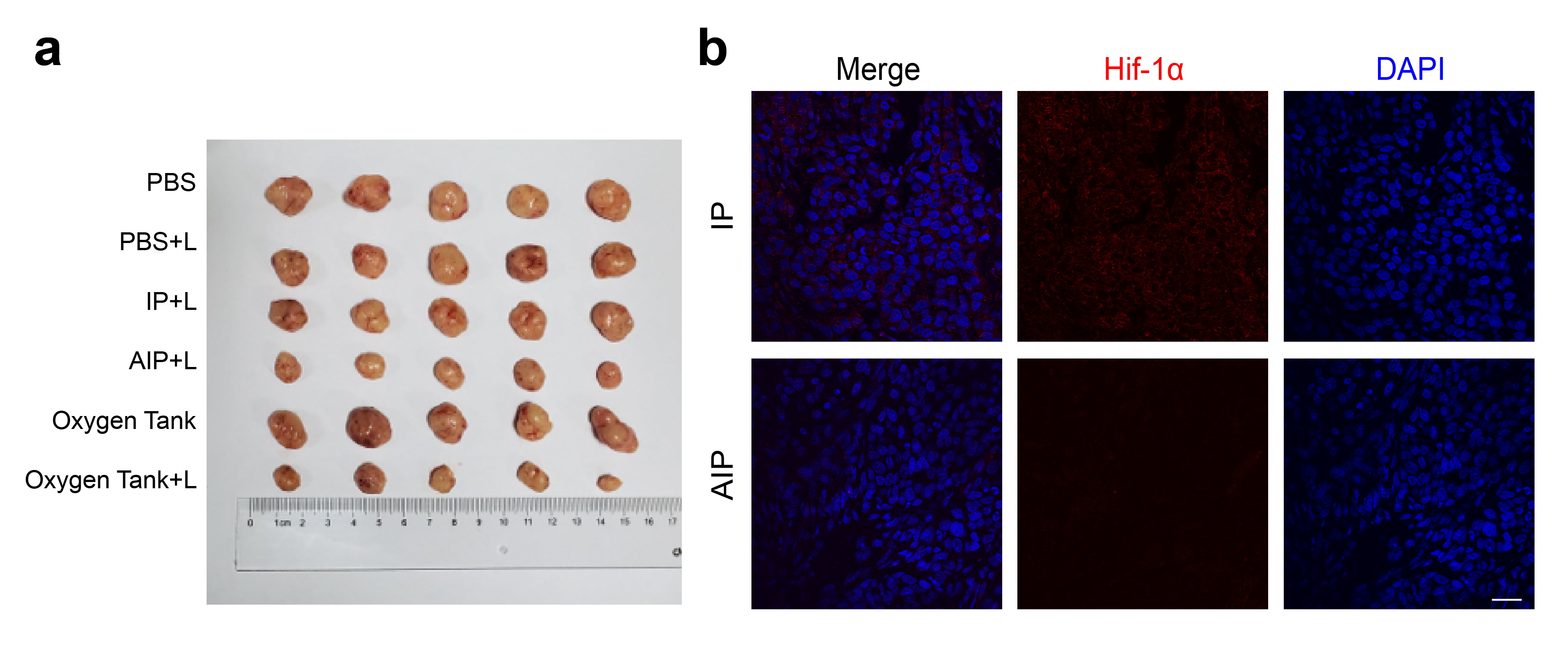


***Figure S9.*** *a) Photograph of tumors of in vivo anti-tumor evaluation (n=5). b) Hif-1α staining tumor sections. The scale bar is 50* *μm.*


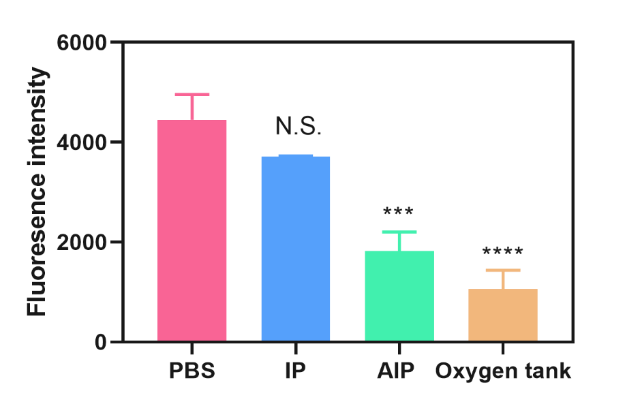


***Figure S10.*** *Quantitative result of Hif-1α staining tumor sections after different treatments (PBS, IP NPs, AIP NPs, and Oxygen Tank).* *Data are showed as mean ± SD (n=3).*

*
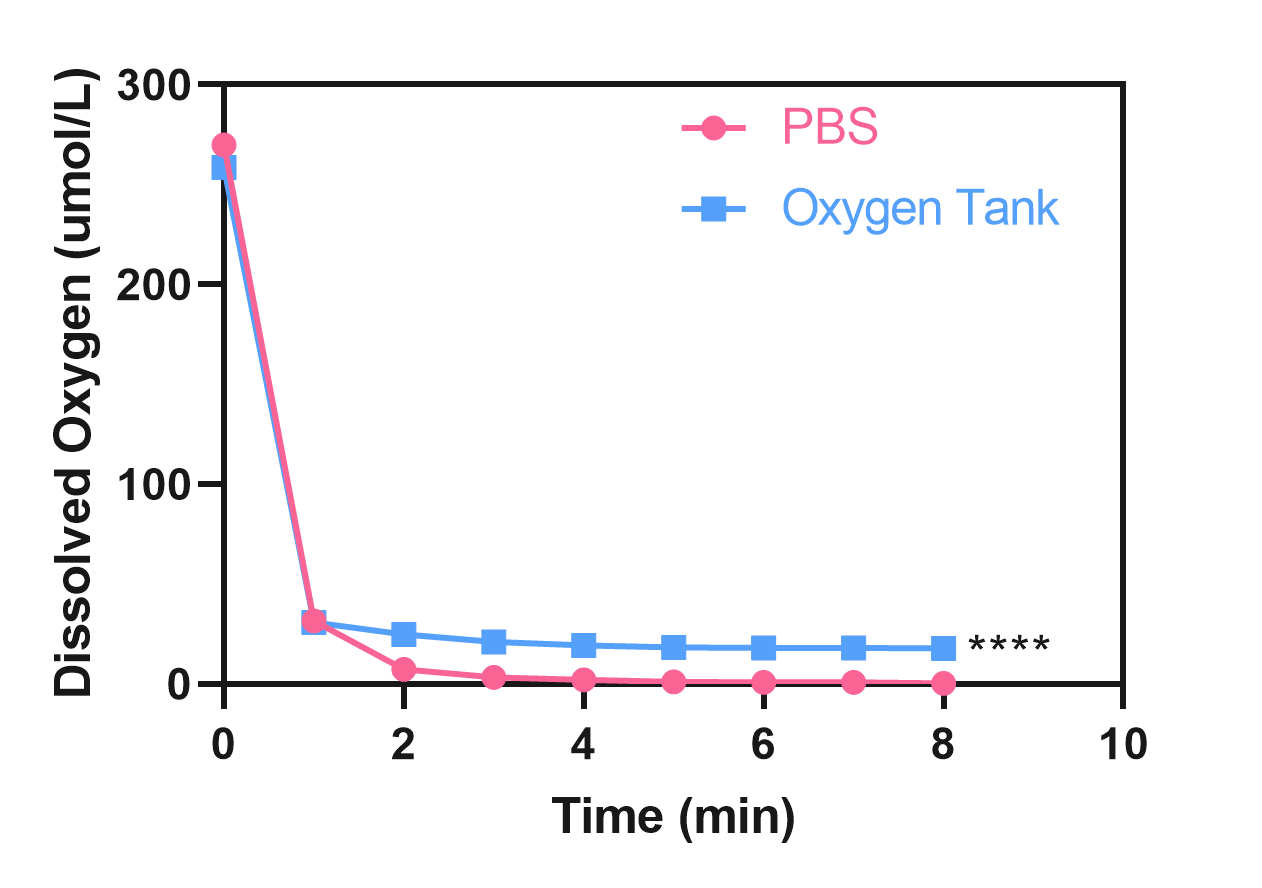
*

***Figure S11.*** *The dissolved oxygen curves in tumor site after different treatments (PBS or Oxygen Tank, 200uL, 100ug/mL IR780, tail vein injection). Start recording once the oxygen probe was inserted into the tumor in vivo. AGS bearing mice were anesthetized during the experiment.*


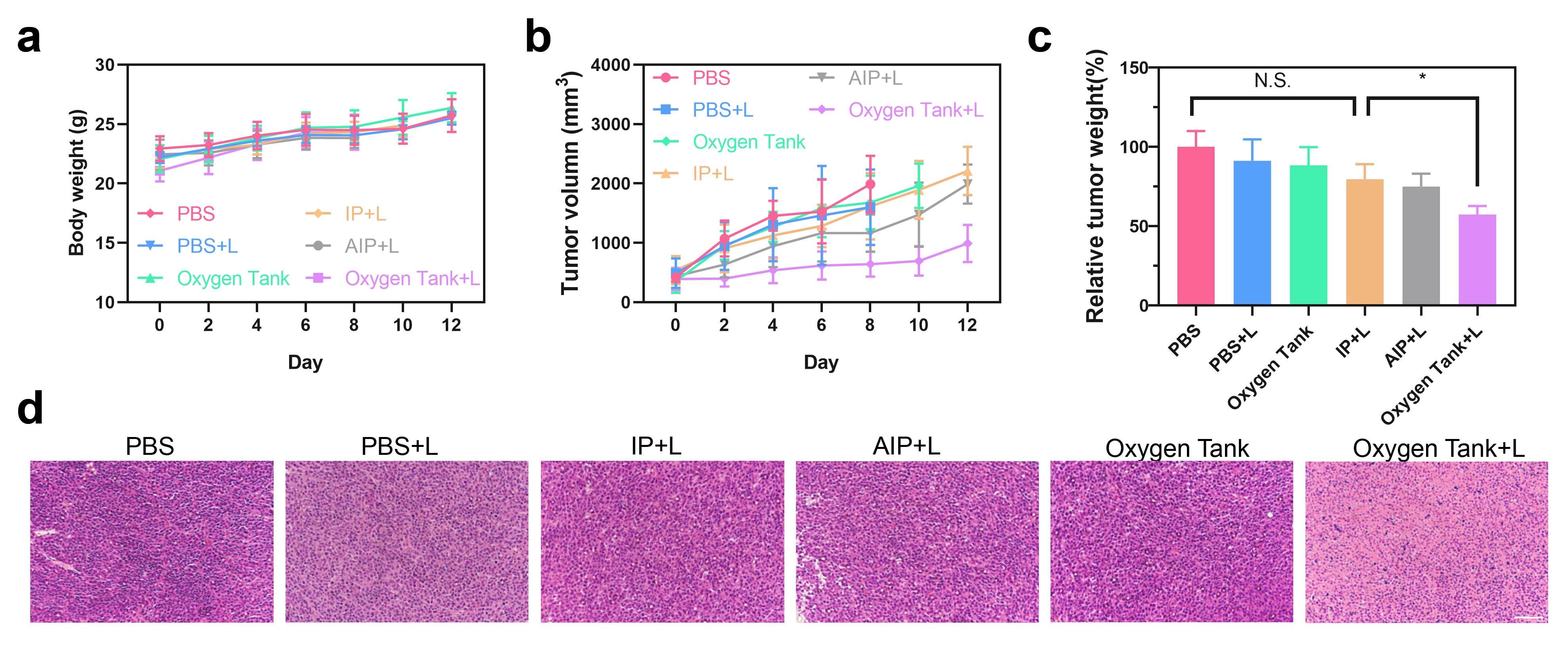


***Figure S12****. In vivo anti-tumor effect of Oxygen Tank in CT26 bearing mice* *(200 μL, 100 μg/mL,* *tail vein injection, n=6). a) The body weight curves. b) The tumor volume curves. c) Weight of tumors. d) H&E staining tumor sections. The scale bar is 200um. Data are showed as mean ± SD. *p<0.05, while N.S. means Not Significant.*


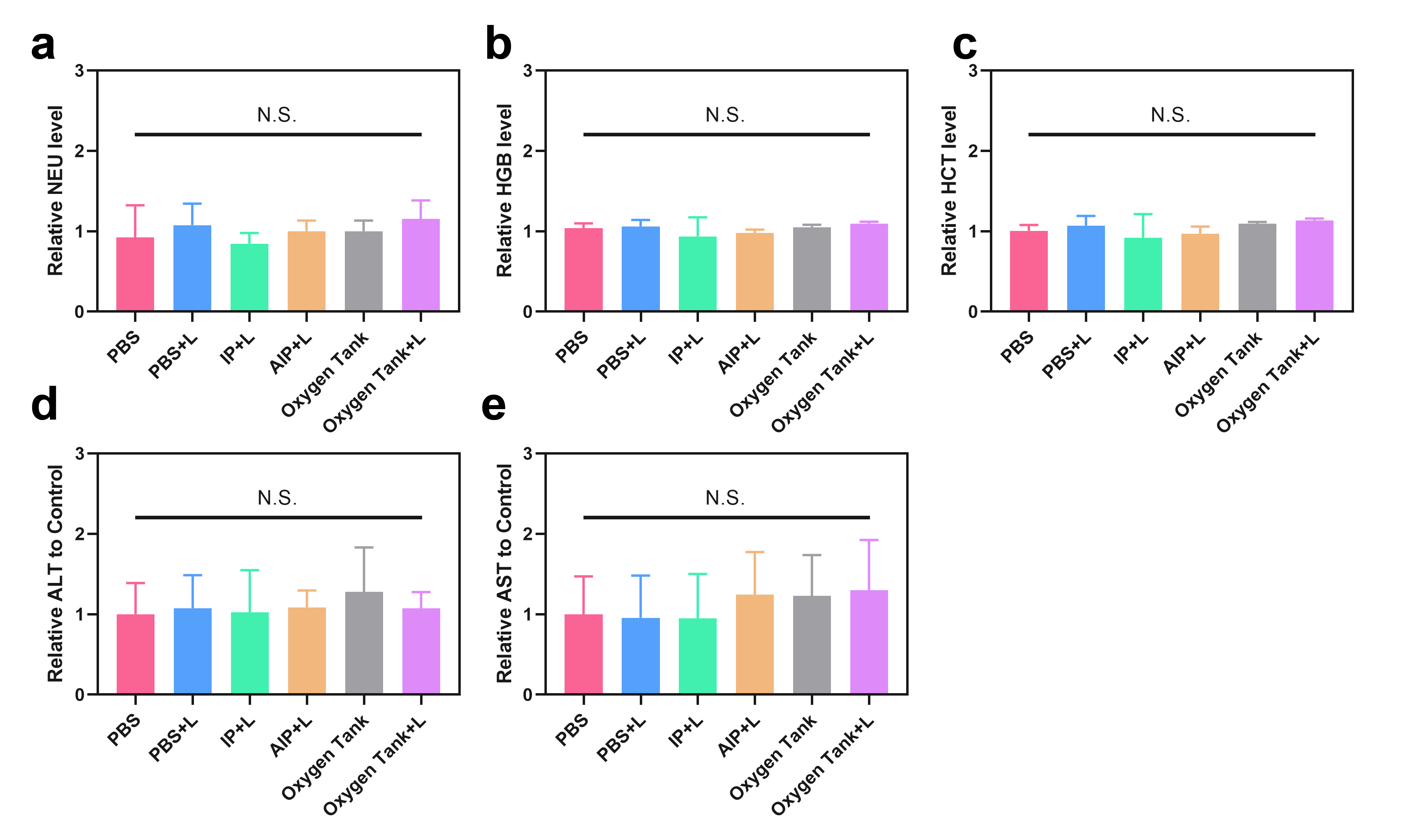
­­

***Figure S13****. Potential long-term in vivo biosafety analysis of Oxygen Tank (200μL, 100μg/mL IR780, tail vein injection). a-c) Hematology assay (NEU: neutrophils; HGB: hemoglobin; PLT: platelets). d-e) Serum biochemical assay (ALT: alanine aminotransferase; AST, aspartate aminotransferase). Data are demonstrated as mean ± SD and analyzed by one-way ANOVA method (n=3). N.S. means Not Significant.*

**Table S1. Peak table of high-performance liquid chromatography result of ATO**

| No. | Retention Time | Area | Height |
| --- | --- | --- | --- |
| 1 | 2.111 | 2138 | 318 |
| 2 | 2.465 | 14402 | 3300 |
| 3 | 2.566 | 7892 | 1601 |
| 4 | 2.856 | 30307 | 9465 |
| 5 | 3.052 | 42923 | 5071 |
| 6 | 3.307 | 453626 | 19686 |
| 7 | 3.797 | 60417 | 4537 |
| 8 | 4.308 | 325153 | 37532 |
| 9 | 4.861 | 28954 | 1911 |
| 10 | 5.288 | 1139 | 878 |
| 11 | 5.693 | 155567 | 11911 |
| 12 | 6.381 | 9865 | 883 |
| 13 | 6.635 | 2300 | 193 |
| 14 | 7.515 | 45646859 | 2203366 |
| 15 | 8.715 | 3007 | 341 |
| 16 | 9.177 | 10494 | 770 |
| SUM | / | 46805260 | 2301764 |

**Table S2. Peak table of high-performance liquid chromatography results of Oxygen Tank.**

| No. | Retention Time | Area | Height |
| --- | --- | --- | --- |
| 1 | 2.405 | 15542 | 5438 |
| 2 | 2.491 | 31841 | 9239 |
| 3 | 2.592 | 82954 | 11427 |
| 4 | 2.783 | 247237 | 39542 |
| 5 | 3.387 | 2139187 | 102404 |
| 6 | 3.798 | 1155713 | 140485 |
| 7 | 4.162 | 2646340 | 367585 |
| 8 | 4.434 | 1229312 | 134296 |
| 9 | 4.785 | 693056 | 74443 |
| 10 | 5.179 | 491298 | 49260 |
| 11 | 5.575 | 362327 | 32811 |
| 12 | 5.988 | 245033 | 20916 |
| 13 | 6.441 | 164432 | 13015 |
| 14 | 6.929 | 89956 | 7533 |
| 15 | 7.500 | 14005405 | 1375286 |
| 16 | 8.297 | 372565 | 16910 |
| 17 | 8.693 | 561713 | 12399 |
| 18 | 10.069 | 65748 | 2922 |
| 19 | 10.458 | 45116 | 2879 |
| 20 | 15.676 | 5828 | 275 |
| 21 | 17.074 | 17930 | 477 |
| 22 | 18.650 | 22038 | 468 |
| 23 | 26.230 | 10040 | 313 |
| SUM | / | 24700612 | 2023041 |
